# Stiffness characteristics analysis of a Biglide industrial parallel robot considering the gravity of mobile platform and links

**DOI:** 10.1038/s41598-023-34214-8

**Published:** 2023-05-05

**Authors:** Jiuliang Guan, Ping Zou, Jilin Xu, Wenjie Wang

**Affiliations:** School of Mechanical Engineering and Automation, Northeastern University, Shenyang, 110819 China

**Keywords:** Engineering, Mechanical engineering

## Abstract

For the machining process of industrial parallel robots, the gravity generated by the weight of mobile platform and links will lead to the deviation of the expected machining trajectory of the tool head. In order to evaluate this deviation and then circumvent it, it is necessary to perform the robotic stiffness model. However, the influence of gravity is seldom considered in the previous stiffness analysis. This paper presents an effective stiffness modeling method for industrial parallel robots considering the link/joint compliance, the mobile platform/link gravity, and the mass center position of each link. First, the external gravity corresponding to each component is determined by the static model under the influence of gravity and mass center position. Then, the corresponding Jacobian matrix of each component is obtained by the kinematic model. Subsequently, the compliance of each component is obtained by cantilever beam theory and FEA-based virtual experiments. In turn, the stiffness model of the whole parallel robot is determined and the Cartesian stiffness matrix of the parallel robot is calculated at several positions. Moreover, the principal stiffness distribution of the tool head in each direction over the main workspace is predicted. Finally, the validity of the stiffness model with gravity is experimentally proved by the comparison of the calculated stiffness and measured stiffness in identical conditions.

## Introduction

In many modern applications, such as manufacturing robots^1,2^, bionic robots^3,4^, medical robots^5,6^ and aeronautical robots^7,8^, the mechanism is subject to external payloads and gravity of its own components which in turn induce deformations causing positioning errors. In order to improve motion accuracy and machining accuracy, stiffness analysis becomes an important solution. Moreover, stiffness analysis is also very important for the design stage of a parallel robot^9^. This is why stiffness analysis has become a research hotspot in recent years.

In terms of stiffness modeling, stiffness modeling methods can be mainly divided into three categories^10^: the finite element analysis method (FEA), the matrix structural analysis method (MSA) and the virtual joint modeling method (VJM). The FEA divides the target model into smaller and simpler connected element modules according to its real dimension and shape. Therefore, the modeling accuracy of FEA is very high, but its calculation cost is also very large. It needs the help of commercial FEA software to complete the modeling of the target model, and in order to ensure that the nodes of the module in the division match the nodes on the model, it needs to re-mesh the finite element model repeatedly. That's why it's expensive to calculate, and the process is time-consuming and tedious. Therefore, the FEA is not suitable for parametric stiffness model, which requires stiffness evaluation of the entire workspace.

The MSA method takes each component of the robot as a structural unit, which is a large compliant element, and combines the main ideas of the FEA to establish stiffness model of the robot. The main idea of this method is to obtain the stiffness matrix of the robot by combining the stiffness matrix of standard elements such as links, joints and frames with matrix analysis method. However, the distortion of this method is that these substructures are regarded as regular shapes, but the stiffness matrix of their standard element cannot accurately describe the actual stiffness of these substructures. Although some scholars have made some innovations on this method^11–13^, this method is still not suitable for the stiffness modeling of robot with the substructure of multi-node element.

In these approaches, the VJM is the most attractive and is adopted in this paper. This method obtains the stiffness model of the robot by establishing the force and deformation mapping relationship between joint space and operation space and applying Jacobian matrix and Hooke's law. And the overall compliance factor comes only from the joints embedded by the virtual springs. This method was first proposed by Salisbury^14^, and then elaborated by Gosselin^15^. Pashkevich et al.^16,17^ considered the compliance of the link on this basis, and described the translational/rotational compliance of the link and the joint as well as the coupling between them by using a 6-dof virtual spring. The method is not only simple to calculate but also reliable in calculation accuracy, so it has been widely popular^18–24^.

However, with the increasing machining accuracy of current industrial processes, the requirements for stiffness models are also increasing, which requires calculations that are both accurate and efficient. Dong et al.^25^ combined FEA and VJM to propose a semi-analytical stiffness modeling method with high computational accuracy, which only performs FEA on some non-standard components in order to avoid excessive computational consumption. However, the method does not consider the influence of the component gravity. Chen et al.^26^ proposed a stiffness model for robots under the action of external gravity. On this basis, Klimchik et al.^27^ proposed a stiffness model for parallel robots under internal and external loads. Lian et al.^28^ proposed a stiffness model for a five-degree-of-freedom parallel robot by the links gravity. Wang et al.^29^ proposed a stiffness model of a five-axis parallel robot by considering the links gravity. However, all these stiffness models consider the gravity of the link as an external load acting on the end-effector without considering the influence of the end-effector gravity and the mass center position of the link. Also, Xu et al.^30^ proposed a stiffness modeling method considering the gravity combined with the deflection superposition principle applied to heavy industrial tandem robots. But this modeling method is not part of the VJM family of methods and has not been applied to parallel robots.

**Figure 1:**
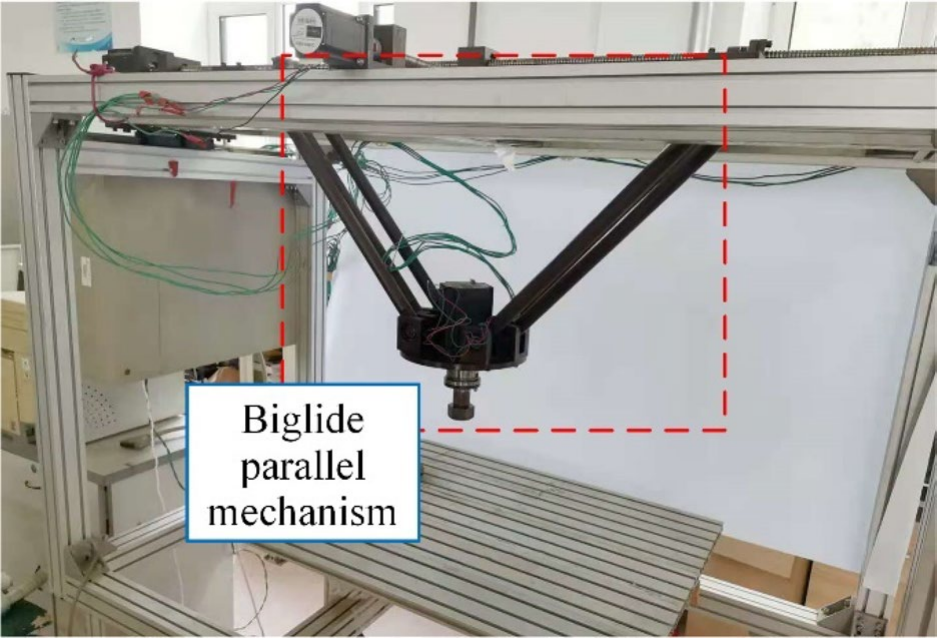
Mechanical structure of the Biglide parallel robot.

In response to the limitations of previous studies and combining their advantages, this study proposes an effective stiffness modeling method for industrial parallel robots. It allows to evaluate the stiffness distribution of an industrial parallel robot in its task workspace considering the influence of the mobile platform/link gravity and the mass center position of each link. Firstly, the model takes into account the compliance of the links/joints by attaching 6-dof virtual springs. Secondly, the model also takes into account the gravity of the links and the mobile platform, as well as the variation of their mass center position in the workspace. Finally, only the compliance matrix of individual irregular components is identified by using the FEA-based virtual experiment to ensure the accuracy and efficiency of the model. In this paper, the Biglide industrial parallel robot in Fig. 1 is used as an example to develop a stiffness model and predict the principal stiffness distribution in the task workspace. The correctness of the model is demonstrated by comparing it with experiments under the same conditions.

## Stiffness modeling procedure of the Biglide parallel robot

**Figure 2:**
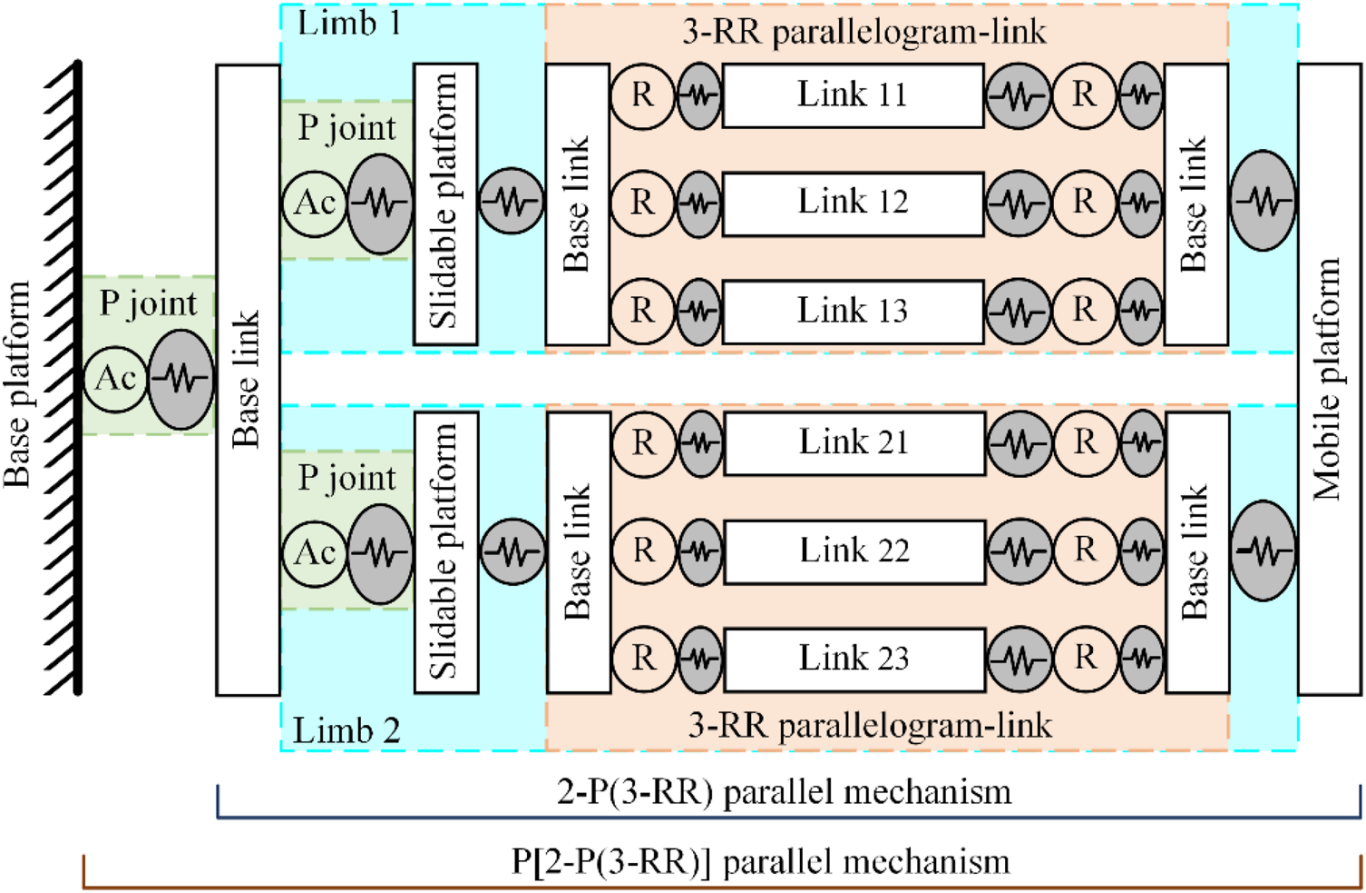
The elastic model of the Biglide parallel robot.

In this section, the stiffness modeling method for the Biglide parallel robot that considers both the compliance of all components and the gravity of moveable components is presented^16–19,26,27^. Each component is either a link/slidable platform or a 1-degree-of-freedom (dof) revolute joint/prismatic joint. The moveable components are composed of the mobile platform and links. The elastic system of the Biglide parallel robot is shown in Fig. 2.

1. Stiffness model of the parallelogram-link (3-RR mechanism)

The 3-RR parallelogram-link (Pl) structure is composed of three links, which has a revolute joint at each end. By considering the effects of the gravity of the mobile platform and the revolute joints on the stiffness of each link, the Cartesian stiffness matrix ${\mathbf{K}}_{{{\mathrm{C}},{\mathrm{Pl}},j}}^{i}$ of each link can be expressed as

1 $$\left\{ {\begin{array}{*{20}l} {{\mathbf{K}}_{{{\mathrm{C}},{\mathrm{Pl}},j}}^{i} = {\mathbf{K}}_{{{\mathrm{C}},{\mathrm{Pl}},j}}^{0,i} - {\mathbf{K}}_{{{\mathrm{C}},{\mathrm{Pl}},j}}^{0,i} \cdot {\mathbf{J}}_{{{\mathrm{q}},{\mathrm{Pl}},j}}^{i} \cdot \left[ {{\mathbf{J}}_{{{\mathrm{q}},{\mathrm{Pl}},j}}^{{i{\mathrm{ T}}}} \cdot \left( {{\mathbf{K}}_{{{\uptheta },{\mathrm{Pl}},j}}^{i} - {\mathbf{K}}_{{{\mathrm{G}},{\mathrm{Pl}},j}}^{i} } \right) \cdot {\mathbf{J}}_{{{\mathrm{q}},{\mathrm{Pl}},j}}^{i} } \right]^{ - 1} \cdot {\mathbf{J}}_{{{\mathrm{q}},{\mathrm{Pl}},j}}^{{i{\mathrm{ T}}}} \cdot {\mathbf{K}}_{{{\mathrm{C}},{\mathrm{Pl}},j}}^{0,i} } \hfill \\ {{\mathbf{K}}_{{{\mathrm{C}},{\mathrm{Pl}},j}}^{0,i} = {\mathbf{J}}_{{{\uptheta },{\mathrm{Pl}},j}}^{{i \, - {\mathrm{T}}}} \cdot \left( {{\mathbf{K}}_{{{\uptheta },{\mathrm{Pl}},j}}^{i} - {\mathbf{K}}_{{{\mathrm{G}},{\mathrm{Pl}},j}}^{i} } \right) \cdot {\mathbf{J}}_{{{\uptheta },{\mathrm{Pl}},j}}^{i \, - 1} } \hfill \\ \end{array} } \right.$$

where ${\mathbf{K}}_{{{\uptheta },{\mathrm{Pl}},j}}^{i}$ is the stiffness matrix of the link, ${\mathbf{K}}_{{{\mathrm{G}},{\mathrm{Pl}},j}}^{i}$ denotes the stiffness matrix due to the gravity of the mobile platform for the link, ${\mathbf{J}}_{{{\uptheta },{\mathrm{Pl}},j}}^{i}$ is the Jacobian matrix of the virtual spring corresponding to the link, ${\mathbf{J}}_{{{\mathrm{q}},{\mathrm{Pl}},j}}^{i}$ is the Jacobian matrix related to the revolute joint, *i* is the number of limbs (*i* = 1, 2), *j* is the number of links in the 3-RR parallelogram-link structure (*j* = 1, 2, 3). For accuracy of expression, *i* and *j* are taken as such throughout the whole paper.

The three links in the parallelogram-link structure are connected in parallel, then the Cartesian stiffness matrix ${\mathbf{K}}_{{{\mathrm{C}},{\mathrm{Pl}}}}^{i}$ of the 3-RR parallelogram-link structure can be expressed as

2 $$ {\mathbf{K}}_{{{\mathrm{C}},{\mathrm{Pl}}}}^{i} = {\mathbf{J}}_{{{\uptheta },{\mathrm{Pl}}}}^{{i \, - {\mathrm{T}}}} \cdot \left( {{\mathbf{K}}_{{{\uptheta },{\mathrm{Pl}}}}^{i} - {\mathbf{K}}_{{{\mathrm{G}},{\mathrm{Pl}}}}^{i} } \right) \cdot {\mathbf{J}}_{{{\uptheta },{\mathrm{Pl}}}}^{i \, - 1} , \, {\mathbf{K}}_{{{\uptheta },{\mathrm{Pl}}}}^{i} - {\mathbf{K}}_{{{\mathrm{G}},{\mathrm{Pl}}}}^{i} = \sum\limits_{j = 1}^{3} {{\mathbf{K}}_{{{\mathrm{C}},{\mathrm{Pl}},j}}^{i \, } } $$

where ${\mathbf{K}}_{{{\uptheta },{\mathrm{Pl}}}}^{i}$ is the stiffness matrix of the 3-RR parallelogram-link structure, ${\mathbf{K}}_{{\mathrm{G,Pl}}}^{i}$ denotes the stiffness matrix due to the gravity of the mobile platform for the 3-RR parallelogram-link structure, ${\mathbf{J}}_{{{\uptheta },{\mathrm{Pl}}}}^{i}$ is the Jacobian matrix of the virtual spring corresponding to the 3-RR parallelogram-link structure.

(2) Stiffness model of the single limb (P(3-RR) mechanism)

In the P(3-RR) mechanism, prismatic joint (actuator), slidable platform (SP) and parallelogram-link structure are connected in series, then the Cartesian stiffness matrix ${\mathbf{K}}_{{\mathrm{C,limb}}}^{i}$ of the single limb can be expressed as

3 $$ \begin{gathered} {\mathbf{K}}_{{{\mathrm{C}},{\mathrm{limb}}}}^{i} = \left( {{\mathbf{K}}_{{{\mathrm{C}},{\mathrm{Ac}}}}^{i \, - 1} + {\mathbf{K}}_{{{\mathrm{C}},{\mathrm{SP}}}}^{i \, - 1} + {\mathbf{K}}_{{{\mathrm{C}},{\mathrm{Pl}}}}^{i \, - 1} } \right)^{ - 1} = {(}{\mathbf{J}}_{{{\uptheta },{\mathrm{Ac}}}}^{i} \cdot K_{{{\uptheta },{\mathrm{Ac}}}}^{ - 1} \cdot {\mathbf{J}}_{{{\uptheta },{\mathrm{Ac}}}}^{{i{\mathrm{ T}}}} + {\mathbf{J}}_{{{\uptheta },{\mathrm{SP}}}}^{i} \cdot \left( {{\mathbf{K}}_{{{\uptheta },{\mathrm{SP}}}}^{i} - {\mathbf{K}}_{{{\mathrm{G}},{\mathrm{SP}}}}^{i} } \right)^{ - 1} \hfill \\ \, \cdot {\mathbf{J}}_{{{\uptheta },{\mathrm{SP}}}}^{{i{\mathrm{ T}}}} + {\mathbf{J}}_{{{\uptheta },{\mathrm{Pl}}}}^{i} \cdot \left( {{\mathbf{K}}_{{{\uptheta },{\mathrm{Pl}}}}^{i} - {\mathbf{K}}_{{{\mathrm{G}},{\mathrm{Pl}}}}^{i} } \right)^{ - 1} \cdot {\mathbf{J}}_{{{\uptheta },{\mathrm{Pl}}}}^{{i{\mathrm{ T}}}} {)}^{ - 1} \hfill \\ \end{gathered} $$

where ${\mathbf{K}}_{{{\mathrm{C}},{\mathrm{Ac}}}}^{i}$ is the Cartesian stiffness matrix of the actuator (lead screw driving system), ${\mathbf{K}}_{{{\mathrm{C}},{\mathrm{SP}}}}^{i}$ is the Cartesian stiffness matrix of the slidable platform, $K_{{{\uptheta },{\mathrm{Ac}}}}$ is the stiffness coefficient of the driving system consisting of the motor and the screw, ${\mathbf{K}}_{{{\uptheta },{\mathrm{SP}}}}^{i}$ is the stiffness matrix of the slidable platform, ${\mathbf{K}}_{{{\mathrm{G}},{\mathrm{SP}}}}^{i}$ denotes the stiffness matrix due to the gravity of the mobile platform and links for the slidable platform, ${\mathbf{J}}_{{{\uptheta },{\mathrm{Ac}}}}^{i}$ is the Jacobian matrix related to the actuator (prismatic joint), ${\mathbf{J}}_{{{\uptheta },{\mathrm{SP}}}}^{i}$ is the Jacobian matrix of the virtual spring corresponding to the slidable platform.

(3) Stiffness model of the parallel limbs (2-P(3-RR) mechanism)

In the 2-P(3-RR) mechanism, the two limbs are connected in parallel and the size of the mobile platform is considered, then the Cartesian stiffness matrix ${\mathbf{K}}_{{{\mathrm{C}},{\mathrm{limb}}}}$ of the 2P(3-RR) mechanism can be expressed as

4 $$ {\mathbf{K}}_{{{\mathrm{C}},{\mathrm{limb}}}} = \sum\limits_{i = 1}^{2} {\left( {{\mathbf{J}}_{{\mathrm{v}}}^{{i \, - {\mathrm{T}}}} \cdot {\mathbf{K}}_{{{\mathrm{C}},{\mathrm{limb}}}}^{i} \cdot {\mathbf{J}}_{{\mathrm{v}}}^{i \, - 1} } \right)} $$

where ${\mathbf{J}}_{{\mathrm{v}}}^{i}$ defines geometrical mapping between end-points of serial limbs and reference point frame (end-effector).

(4) Stiffness model of the whole Biglide parallel robot (P[2-P(3-RR)] mechanism).

In the P[2-P(3-RR)] mechanism, prismatic joint (actuator) and 2-P(3-RR) mechanism are connected in series in the Biglide parallel robot, then the Cartesian stiffness matrix **K**_C_ of the Biglide parallel robot can be expressed as

5 $$ {\mathbf{K}}_{{\mathrm{C}}} = \left( {{\mathbf{J}}_{{{\uptheta },{\mathrm{Ac}}}}^{0} \cdot K_{{{\uptheta },{\mathrm{Ac}}}}^{ - 1} \cdot {\mathbf{J}}_{{{\uptheta },{\mathrm{Ac}}}}^{{\mathrm{0 T}}} + {\mathbf{K}}_{{{\mathrm{C}},{\mathrm{limb}}}}^{ - 1} } \right)^{ - 1} $$

where ${\mathbf{J}}_{{{\uptheta },{\mathrm{Ac}}}}^{0}$ is the Jacobian matrix corresponding to the first prismatic joint.

Moreover, in order to solve the above equations, the static and kinematic analysis of the Biglide parallel robot is required.

## Static analysis of the Biglide parallel robot

In this section, the respective static models are developed at each component of the Biglide parallel robot, taking into account the gravity generated by the weight of the mobile platform and the moveable links^26–29^. The mathematical equation of the static force/torque corresponding to each component is derived, and thus the gravity received at the component is obtained.

### Coordinate system of the Biglide parallel robot

The mechanical structure illustration of the Biglide parallel robot is offered in Fig. 1. The Biglide parallel robot is a two degree-of-freedom (2-dof) parallel mechanism (PM) and consist of two modular parallelogram-links connected to the mobile platform (MP) and two sliding units which are actuated via lead screw system, where the sliding units are installed on the upper fixed platform. The Biglide parallel robot can generate two translations in the horizontal/vertical directions within a single plane, and the single moving plane can also move along its normal direction.

**Figure 3:**
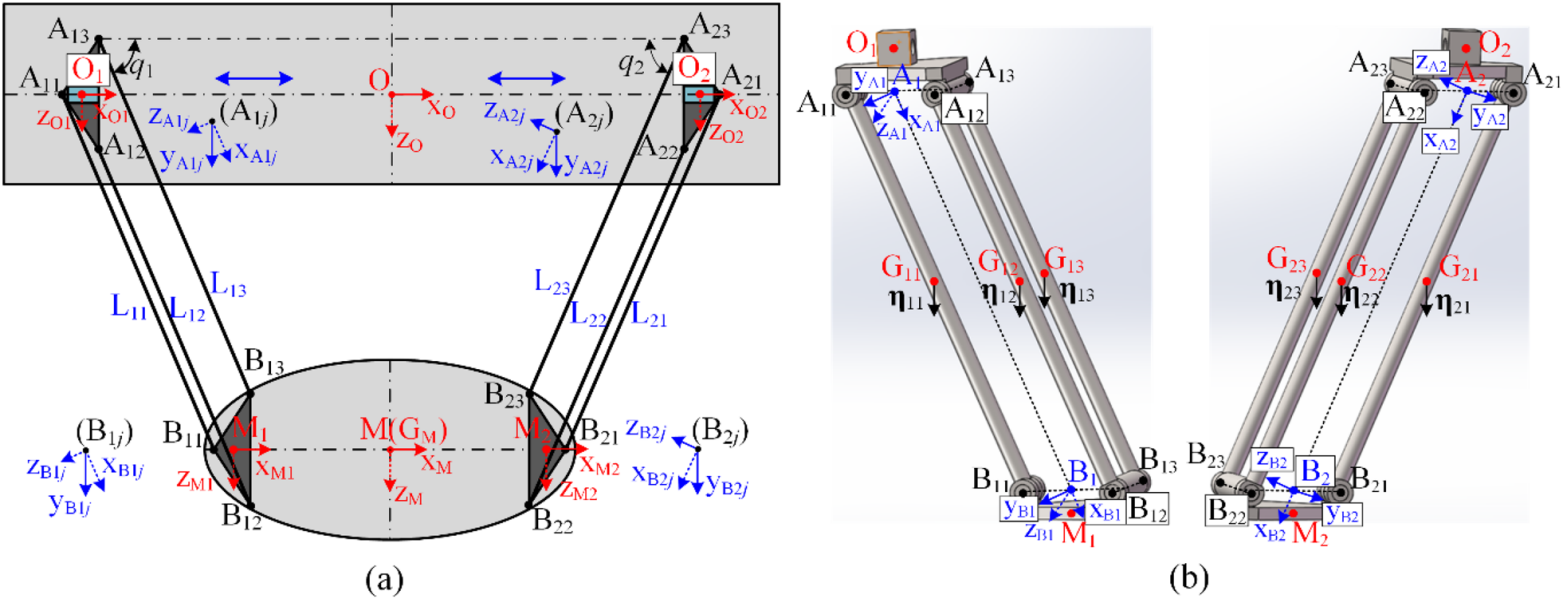
Coordinate system: (**a**) Biglide parallel robot; (**b**) Left and right limbs.

To convenience the calculation, a sequence of coordinate systems is attached to each substructure of the Biglide parallel robot. These coordinate systems are expressed as {O}, {O_*i*_}, {A_*i*_}, {A_*ij*_}, {B_*ij*_}, {B_*i*_}, {M_*i*_} and {M} respectively (*i* = 1, 2; *j* = 1, 2, 3), as shown in Fig. 3. The origin and directions are determined by the joint axis and the center line of the substructures. It is worth noting that the direction of the coordinate systems {A_*ij*_} and {B_*ij*_} are parallel to the direction of {A_*i*_} and {B_*i*_}, respectively, except that the origin of {A_*ij*_} and {B_*ij*_} are the points A_*ij*_ and B_*ij*_, respectively. Moreover, a_*i*_ represents the length of OO_*i*_ (a_*i*_ ∈ [200, 550] mm); *l* and *L* represent the length of MM_*i*_ and the link (L_*ij*_), respectively; *q*_*i*_ denotes the angle between A_*i*1_B_*i*1_ and O_1_O_2_ (*q*_1_ = *q*_2_ = *q*), *d* is half the length of B_*i*2_B_*i*3_, O_*i*_A_*i*_ (length *l*_1_) and B_*i*_M_*i*_ (length *l*_2_) are always parallel to the axis z_o_, *h* represents the distance from point B_*i*_ to B_*i*2_B_*i*3_ or the length of B_*i*_ B_*i*1_. The orientation matrices of {O}, {O_*i*_}, {A_*ij*_}, {B_*ij*_}, {M_*i*_} and {M} relative to {O} can be expressed as

6 $$\left\{ {\begin{array}{*{20}l}    {{\mathbf{R}}_{{{\mathrm{O1}}}} {\mathrm{ = }}{\mathbf{R}}_{{\mathrm{O}}}  \cdot {\mathbf{R}}\left( {y_{{\mathrm{O}}} ,0} \right)} \hfill  \\    {{\mathbf{R}}_{{{\mathrm{A1}}j}}  = {\mathbf{R}}_{{{\mathrm{O1}}}}  \cdot {\mathbf{R}}\left( {y_{{{\mathrm{O1}}}} , - q_{1} } \right)} \hfill  \\    {{\mathbf{R}}_{{{\mathrm{B1}}j}} {\mathrm{ = }}{\mathbf{R}}_{{{\mathrm{A1}}j}}  \cdot {\mathbf{R}}\left( {y_{{{\mathrm{A1}}j}} ,0} \right)} \hfill  \\    {{\mathbf{R}}_{{{\mathrm{M1}}}} {\mathrm{ = }}{\mathbf{R}}_{{{\mathrm{B1}}j}}  \cdot {\mathbf{R}}\left( {y_{{{\mathrm{B1}}j}} ,q_{1} } \right)} \hfill  \\    {{\mathbf{R}}_{{\mathrm{M}}} {\mathrm{ = }}{\mathbf{R}}_{{{\mathrm{M1}}}}  \cdot {\mathbf{R}}\left( {y_{{{\mathrm{M1}}}} ,0} \right)} \hfill  \\   \end{array} } \right.,{\mathrm{ }}\left\{ {\begin{array}{*{20}l}    {{\mathbf{R}}_{{{\mathrm{O2}}}} {\mathrm{ = }}{\mathbf{R}}_{{\mathrm{O}}}  \cdot {\mathbf{R}}\left( {y_{{\mathrm{O}}} ,0} \right)} \hfill  \\    {{\mathbf{R}}_{{{\mathrm{A2}}j}}  = {\mathbf{R}}_{{{\mathrm{O2}}}}  \cdot {\mathbf{R}}\left( {y_{{{\mathrm{O2}}}} ,q_{2}  - {{\uppi }}} \right)} \hfill  \\    {{\mathbf{R}}_{{{\mathrm{B2}}j}} {\mathrm{ = }}{\mathbf{R}}_{{{\mathrm{A2}}j}}  \cdot {\mathbf{R}}\left( {y_{{{\mathrm{A2}}j}} ,0} \right)} \hfill  \\    {{\mathbf{R}}_{{{\mathrm{M2}}}} {\mathrm{ = }}{\mathbf{R}}_{{{\mathrm{B2}}j}}  \cdot {\mathbf{R}}\left( {y_{{{\mathrm{B2}}j}} ,{{\uppi }} - q_{2} } \right)} \hfill  \\    {{\mathbf{R}}_{{\mathrm{M}}}  = {\mathbf{R}}_{{\mathrm M{2}}}  \cdot {\mathbf{R}}\left( {y_{{{\mathrm{M2}}}} ,0} \right)} \hfill  \\   \end{array} } \right.$$

where **R**(.) represents a 3 × 3 rotation matrix that rotates the corresponding angle around the corresponding axis, **R**_O_ is an identity matrix, and **R**_*n*_ is the orientation matrix of the coordinate system {O_*n*_} with respect to the coordinate system {O}.

### Static equilibrium equation of points M_***i***_

In order to evaluate the influence of the mobile platform weight at points M_1_ and M_2_, it is necessary to analysis the static force/torque balance at points M_1_ and M_2_, as shown in Fig. 3. The static equilibrium equations at point M can be displayed as

7 $$ \left\{ {\begin{array}{*{20}l} {m_{{{\mathrm{MP}}}} \cdot g \cdot {\mathbf{z}} = {\mathbf{R}}_{{{\mathrm{M1}}}} \cdot {\mathbf{f}}_{{{\mathrm{M1}}}} + {\mathbf{R}}_{{{\mathrm{M2}}}} \cdot {\mathbf{f}}_{{{\mathrm{M2}}}} } \hfill \\ {m_{{{\mathrm{MP}}}} \cdot g \cdot \left( {{\mathbf{R}}_{{\mathrm{M}}} \cdot {\mathbf{r}}_{{{\mathrm{GM}}}}^{{\mathrm{M}}} } \right) \times {\mathbf{z}} = \left\{ {\begin{array}{*{20}l} {\left( {{\mathbf{R}}_{{\mathrm{M}}} \cdot {\mathbf{r}}_{{{\mathrm{M1}}}}^{{\mathrm{M}}} } \right) \times \left( {{\mathbf{R}}_{{{\mathrm{M1}}}} \cdot {\mathbf{f}}_{{{\mathrm{M1}}}} } \right) + {\mathbf{R}}_{{{\mathrm{M1}}}} \cdot {{\varvec{\uptau}}}_{{{\mathrm{M1}}}} } \hfill \\ { + \left( {{\mathbf{R}}_{{\mathrm{M}}} \cdot {\mathbf{r}}_{{{\mathrm{M2}}}}^{{\mathrm{M}}} } \right) \times \left( {{\mathbf{R}}_{{{\mathrm{M2}}}} \cdot {\mathbf{f}}_{{{\mathrm{M2}}}} } \right) + {\mathbf{R}}_{{{\mathrm{M2}}}} \cdot {{\varvec{\uptau}}}_{{{\mathrm{M2}}}} } \hfill \\ \end{array} } \right.} \hfill \\ \end{array} } \right. $$

where

$$ \begin{gathered} {\mathbf{z}} = \left[ {\begin{array}{*{20}c} 0 & 0 & 1 \\ \end{array} } \right]^{{\mathrm{T}}} , \, {\mathbf{f}}_{{{\mathrm{M1}}}} = \left[ {\begin{array}{*{20}c} {f_{{{\mathrm{M1}},x}} } & {f_{{{\mathrm{M1}},y}} } & {f_{{{\mathrm{M1}},z}} } \\ \end{array} } \right]^{{\mathrm{T}}} , \, {{\varvec{\uptau}}}_{{{\mathrm{B1}}}} = \left[ {\begin{array}{*{20}c} {\tau_{{{\mathrm{M1}},x}} } & {\tau_{{{\mathrm{M1}},y}} } & {\tau_{{{\mathrm{M1}},z}} } \\ \end{array} } \right]^{{\mathrm{T}}} , \hfill \\ {\mathbf{f}}_{{{\mathrm{M2}}}} = \left[ {\begin{array}{*{20}c} {f_{{{\mathrm{M2,}}x}} } & {f_{{{\mathrm{M2,}}y}} } & {f_{{{\mathrm{M2,}}z}} } \\ \end{array} } \right]^{{\mathrm{T}}} , \, {{\varvec{\uptau}}}_{{{\mathrm{M2}}}} = \left[ {\begin{array}{*{20}c} {\tau_{{{\mathrm{M2,}}x}} } & {\tau_{{{\mathrm{M2,}}y}} } & {\tau_{{{\mathrm{M2,}}z}} } \\ \end{array} } \right]^{{\mathrm{T}}} {;} \hfill \\ \end{gathered} $$

$m_{{{\mathrm{MP}}}}$ is the mass of the mobile platform and its fixed motor, G_M_ is the mass center of the mobile platform and coincides with the geometric center M of the mobile platform, **z** denotes the unit vector vertically downward; ${\mathbf{f}}_{{{\mathrm{M1}}}}$ and ${{\varvec{\uptau}}}_{{{\mathrm{M1}}}}$ denote the reaction force and torque applied at point B_1_, ${\mathbf{f}}_{{{\mathrm{M2}}}}$ and ${{\varvec{\uptau}}}_{{{\mathrm{M2}}}}$ denote the reaction force and torque applied at point M_2_; ${\mathbf{r}}_{{{\mathrm{GM}}}}^{{\mathrm{M}}}$ denotes the position vector from point M to point G_M_ expressed in the coordinate system {M}, ${\mathbf{r}}_{{{\mathrm{M1}}}}^{{\mathrm{M}}}$ and ${\mathbf{r}}_{{{\mathrm{M2}}}}^{{\mathrm{M}}}$ denote the position vector from point M to point M_*i*_ expressed in the coordinate system {M}, respectively.

According to the decoupling of Eq. (7), the reaction force acting at points M_1_ and M_2_ can be obtained

8 $$ {{\varvec{\uprho}}}_{{{\mathrm{M1}}}} = \frac{{1}}{{2}}{\mathbf{J}}_{{\mathrm{W,M1}}}^{{\mathrm{T}}} \cdot {{\varvec{\upeta}}}_{{{\mathrm{GM}},{1}}} , \, {{\varvec{\uprho}}}_{{{\mathrm{M2}}}} = \frac{{1}}{{2}}{\mathbf{J}}_{{\mathrm{W,M2}}}^{{\mathrm{T}}} \cdot {{\varvec{\upeta}}}_{{{\mathrm{GM}},{2}}} $$

where

$$ {{\varvec{\upeta}}}_{{{\mathrm{GM}},{1}}} = \frac{1}{2}m_{{{\mathrm{MP}}}} \cdot g \cdot \left[ {\begin{array}{*{20}c} {\mathbf{z}} \\ {\left( {{\mathbf{R}}_{{\mathrm{M}}} \cdot {\mathbf{r}}_{{{\mathrm{GM}}}}^{{\mathrm{M}}} } \right) \times {\mathbf{z}}} \\ \end{array} } \right], \, {{\varvec{\uprho}}}_{{{\mathrm{M1}}}} = \left[ {\begin{array}{*{20}c} {{\mathbf{f}}_{{{\mathrm{M1}}}} } \\ {{{\varvec{\uptau}}}_{{{\mathrm{M1}}}} } \\ \end{array} } \right], \, {\mathbf{W}}_{{{\mathrm{M1}}}} = \left[ {\begin{array}{*{20}c} {{\mathbf{R}}_{{{\mathrm{M1}}}} } & {{\mathbf{s}}\left( {{\mathbf{r}}_{{{\mathrm{M1}}}} } \right) \cdot {\mathbf{R}}_{{{\mathrm{M1}}}} } \\ {\mathbf{0}} & {{\mathbf{R}}_{{{\mathrm{M1}}}} } \\ \end{array} } \right], \, {\mathbf{r}}_{{{\mathrm{M1}}}} = {\mathbf{R}}_{{\mathrm{M}}} \cdot {\mathbf{r}}_{{{\mathrm{M1}}}}^{{\mathrm{M}}} {;} $$

$$ {{\varvec{\upeta}}}_{{{\mathrm{GM}},{2}}} = \frac{1}{2}m_{{{\mathrm{MP}}}} \cdot g \cdot \left[ {\begin{array}{*{20}c} {\mathbf{z}} \\ {\left( {{\mathbf{R}}_{{\mathrm{M}}} \cdot {\mathbf{r}}_{{{\mathrm{GM}}}}^{{\mathrm{M}}} } \right) \times {\mathbf{z}}} \\ \end{array} } \right], \, {{\varvec{\uprho}}}_{{{\mathrm{M2}}}} = \left[ {\begin{array}{*{20}c} {{\mathbf{f}}_{{{\mathrm{M2}}}} } \\ {{{\varvec{\uptau}}}_{{{\mathrm{M2}}}} } \\ \end{array} } \right], \, {\mathbf{W}}_{{{\mathrm{M2}}}} = \left[ {\begin{array}{*{20}c} {{\mathbf{R}}_{{{\mathrm{M2}}}} } & {{\mathbf{s}}\left( {{\mathbf{r}}_{{{\mathrm{M2}}}} } \right) \cdot {\mathbf{R}}_{{{\mathrm{M2}}}} } \\ {\mathbf{0}} & {{\mathbf{R}}_{{{\mathrm{M2}}}} } \\ \end{array} } \right], \, {\mathbf{r}}_{{{\mathrm{M2}}}} = {\mathbf{R}}_{{\mathrm{M}}} \cdot {\mathbf{r}}_{{{\mathrm{M2}}}}^{{\mathrm{M}}} {;} $$

${{\varvec{\uprho}}}_{{{\mathrm{M1}}}}$ and ${{\varvec{\uprho}}}_{{{\mathrm{M2}}}}$ denote the reaction wrench applied at points M_1_ and M_2_, respectively; ${{\varvec{\upeta}}}_{{{\mathrm{GM}},{1}}}$ and ${{\varvec{\upeta}}}_{{{\mathrm{GM}},{2}}}$ denote the equivalent split gravitational wrench of the mobile platform for point M_1_ and M_2_ applied at point M, respectively; ${\mathbf{s}}\left( {{\mathbf{r}}_{{{\mathrm{M1}}}} } \right)$/${\mathbf{s}}\left( {{\mathbf{r}}_{{{\mathrm{M2}}}} } \right)$ represents the skew-matrix of the position vector ${\mathbf{r}}_{{{\mathrm{M1}}}}$/${\mathbf{r}}_{{{\mathrm{M2}}}}$, ${\mathbf{J}}_{{\mathrm{W,M1}}}$/${\mathbf{J}}_{{\mathrm{W,M2}}}$ denotes the adjoint transformation matrix, which can be obtained by the screw theory^31,32^.

### Static equilibrium equation of joints B_***ij***_

In order to evaluate the influence of the mobile platform weight on the joint B_*ij*_, it is necessary to analysis the static force/torque balance at point B_*ij*_, as shown in Fig. 3b. The static equilibrium equations at point M_*i*_ can be displayed as

9 $$ \left\{ {\begin{array}{*{20}l} {\frac{1}{2}m_{{{\mathrm{MP}}}} \cdot g \cdot {\mathbf{z}} = {\mathbf{R}}_{{{\mathrm{B}}i{1}}} \cdot {\mathbf{f}}_{{{\mathrm{B}}i{1}}} { + }{\mathbf{R}}_{{{\mathrm{B}}i{2}}} \cdot {\mathbf{f}}_{{{\mathrm{B}}i{2}}} { + }{\mathbf{R}}_{{{\mathrm{B}}i{3}}} \cdot {\mathbf{f}}_{{{\mathrm{B}}i{3}}} } \hfill \\ {\frac{1}{2}m_{{{\mathrm{MP}}}} \cdot g \cdot \left( {{\mathbf{R}}_{{\mathrm{M}}} \cdot {\mathbf{r}}_{{{\mathrm{GM}}}}^{{\mathrm{M}}} } \right) \times {\mathbf{z}} = \left\{ {\begin{array}{*{20}l} {\left( {{\mathbf{R}}_{{\mathrm{M}}} \cdot {\mathbf{r}}_{{{\mathrm{M}}i}}^{{\mathrm{M}}} + {\mathbf{R}}_{{{\mathrm{M}}i}} \cdot {\mathbf{r}}_{{{\mathrm{B}}i{1}}}^{{{\mathrm{M}}i}} } \right) \times \left( {{\mathbf{R}}_{{{\mathrm{B}}i{1}}} \cdot {\mathbf{f}}_{{{\mathrm{B}}i{1}}} } \right) + {\mathbf{R}}_{{{\mathrm{B}}i{1}}} \cdot {{\varvec{\uptau}}}_{{{\mathrm{B}}i{1}}} } \hfill \\ {{ + }\left( {{\mathbf{R}}_{{\mathrm{M}}} \cdot {\mathbf{r}}_{{{\mathrm{M}}i}}^{{\mathrm{M}}} + {\mathbf{R}}_{{{\mathrm{M}}i}} \cdot {\mathbf{r}}_{{{\mathrm{B}}i{2}}}^{{{\mathrm{M}}i}} } \right) \times \left( {{\mathbf{R}}_{{{\mathrm{B}}i{2}}} \cdot {\mathbf{f}}_{{{\mathrm{B}}i{2}}} } \right) + {\mathbf{R}}_{{{\mathrm{B}}i{2}}} \cdot {{\varvec{\uptau}}}_{{{\mathrm{B}}i{2}}} } \hfill \\ {{ + }\left( {{\mathbf{R}}_{{\mathrm{M}}} \cdot {\mathbf{r}}_{{{\mathrm{M}}i}}^{{\mathrm{M}}} + {\mathbf{R}}_{{{\mathrm{M}}i}} \cdot {\mathbf{r}}_{{{\mathrm{B}}i{3}}}^{{{\mathrm{M}}i}} } \right) \times \left( {{\mathbf{R}}_{{{\mathrm{B}}i{3}}} \cdot {\mathbf{f}}_{{{\mathrm{B}}i{3}}} } \right) + {\mathbf{R}}_{{{\mathrm{B}}i{3}}} \cdot {{\varvec{\uptau}}}_{{{\mathrm{B}}i{3}}} } \hfill \\ \end{array} } \right.} \hfill \\ \end{array} } \right. $$

where

$$ \begin{gathered} {\mathbf{f}}_{{{\mathrm{B}}i{1}}} = \left[ {\begin{array}{*{20}c} {f_{{{\mathrm{B}}i{1,}x}} } & {f_{{{\mathrm{B}}i{1,}y}} } & {f_{{{\mathrm{B}}i{1,}z}} } \\ \end{array} } \right]^{{\mathrm{T}}} , \, {{\varvec{\uptau}}}_{{{\mathrm{B}}i{1}}} = \left[ {\begin{array}{*{20}c} {\tau_{{{\mathrm{B}}i{1,}x}} } & {\tau_{{{\mathrm{B}}i{1,}y}} } & {\tau_{{{\mathrm{B}}i{1,}z}} } \\ \end{array} } \right]^{{\mathrm{T}}} {;} \hfill \\ {\mathbf{f}}_{{{\mathrm{B}}i{2}}} = \left[ {\begin{array}{*{20}c} {f_{{{\mathrm{B}}i{2,}x}} } & {f_{{{\mathrm{B}}i{2,}y}} } & {f_{{{\mathrm{B}}i{2,}z}} } \\ \end{array} } \right]^{{\mathrm{T}}} , \, {{\varvec{\uptau}}}_{{{\mathrm{B}}i{2}}} = \left[ {\begin{array}{*{20}c} {\tau_{{{\mathrm{B}}i{2,}x}} } & {\tau_{{{\mathrm{B}}i{2,}y}} } & {\tau_{{{\mathrm{B}}i{2,}z}} } \\ \end{array} } \right]^{{\mathrm{T}}} {;} \hfill \\ {\mathbf{f}}_{{{\mathrm{B}}i{3}}} = \left[ {\begin{array}{*{20}c} {f_{{{\mathrm{B}}i{3,}x}} } & {f_{{{\mathrm{B}}i{3,}y}} } & {f_{{{\mathrm{B}}i{3,}z}} } \\ \end{array} } \right]^{{\mathrm{T}}} , \, {{\varvec{\uptau}}}_{{{\mathrm{B}}i{3}}} = \left[ {\begin{array}{*{20}c} {\tau_{{{\mathrm{B}}i{3,}x}} } & {\tau_{{{\mathrm{B}}i{3,}y}} } & {\tau_{{{\mathrm{B}}i{3,}z}} } \\ \end{array} } \right]^{{\mathrm{T}}} {;} \hfill \\ \end{gathered} $$

${\mathbf{f}}_{{{\mathrm{B}}i{1}}}$ and ${{\varvec{\uptau}}}_{{{\mathrm{B}}i{1}}}$ denote the reaction force and torque applied at point B_*i*1_, ${\mathbf{f}}_{{{\mathrm{B}}i{2}}}$ and ${{\varvec{\uptau}}}_{{{\mathrm{B}}i{2}}}$ denote the reaction force and torque applied at point B_*i*2_, ${\mathbf{f}}_{{{\mathrm{B}}i{3}}}$ and ${{\varvec{\uptau}}}_{{{\mathrm{B}}i{3}}}$ denote the reaction force and torque applied at point B_*i*3_; ${\mathbf{r}}_{{{\mathrm{B}}i{1}}}^{{{\mathrm{M}}i}}$/${\mathbf{r}}_{{{\mathrm{B}}i{2}}}^{{{\mathrm{M}}i}}$/${\mathbf{r}}_{{{\mathrm{B}}i{3}}}^{{{\mathrm{M}}i}}$ denotes the position vector from point M_*i*_ to point B_*i*1_/ B_*i*2_/ B_*i*3_ expressed in the coordinate system {M_*i*_}.

According to the decoupling of Eq. (9), the reaction force acting on the joints B_*i*1_, B_*i*2_ and B_*i*3_ can be obtained

10 $$ {{\varvec{\uprho}}}_{{{\mathrm{B}}i{1}}} { = }{\mathbf{J}}_{{{\mathrm{W,B}}i{1}}}^{{\mathrm{T}}} \cdot {{\varvec{\upeta}}}_{{{\mathrm{B}}i{1}}} , \, {{\varvec{\uprho}}}_{{{\mathrm{B}}i{2}}} { = }{\mathbf{J}}_{{{\mathrm{W,B}}i2}}^{{\mathrm{T}}} \cdot {{\varvec{\upeta}}}_{{{\mathrm{B}}i{2}}} , \, {{\varvec{\uprho}}}_{{{\mathrm{B}}i{3}}} { = }{\mathbf{J}}_{{{\mathrm{W,B}}i3}}^{{\mathrm{T}}} \cdot {{\varvec{\upeta}}}_{{{\mathrm{B}}i{3}}} $$

where

$$ \begin{gathered} {{\varvec{\upeta}}}_{{{\mathrm{B}}i{1}}} = \frac{1}{3}{{\varvec{\upeta}}}_{{{\mathrm{GM}},i}} , \, {{\varvec{\uprho}}}_{{{\mathrm{B}}i{1}}} = \left[ {\begin{array}{*{20}c} {{\mathbf{f}}_{{{\mathrm{B}}i1}} } \\ {{{\varvec{\uptau}}}_{{{\mathrm{B}}i1}} } \\ \end{array} } \right], \, {\mathbf{J}}_{{{\mathrm{W,B}}i{1}}} = \left[ {\begin{array}{*{20}c} {{\mathbf{R}}_{{{\mathrm{B}}i{1}}} } & {{\mathbf{s}}\left( {{\mathbf{r}}_{{{\mathrm{B}}i{1}}} } \right) \cdot {\mathbf{R}}_{{{\mathrm{B}}i{1}}} } \\ {\mathbf{0}} & {{\mathbf{R}}_{{{\mathrm{B}}i{1}}} } \\ \end{array} } \right], \, {\mathbf{r}}_{{{\mathrm{B}}i{1}}} = {\mathbf{r}}_{{{\mathrm{M}}i}} + {\mathbf{R}}_{{{\mathrm{M}}i}} \cdot {\mathbf{r}}_{{{\mathrm{B}}i{1}}}^{{{\mathrm{M}}i}} {;} \hfill \\ {{\varvec{\upeta}}}_{{{\mathrm{B}}i{2}}} = \frac{1}{3}{{\varvec{\upeta}}}_{{{\mathrm{GM}},i}} , \, {{\varvec{\uprho}}}_{{{\mathrm{B}}i{2}}} = \left[ {\begin{array}{*{20}c} {{\mathbf{f}}_{{{\mathrm{B}}i2}} } \\ {{{\varvec{\uptau}}}_{{{\mathrm{B}}i2}} } \\ \end{array} } \right], \, {\mathbf{J}}_{{{\mathrm{W,B}}i2}} = \left[ {\begin{array}{*{20}c} {{\mathbf{R}}_{{{\mathrm{B}}i{2}}} } & {{\mathbf{s}}\left( {{\mathbf{r}}_{{{\mathrm{B}}i{2}}} } \right) \cdot {\mathbf{R}}_{{{\mathrm{B}}i{2}}} } \\ {\mathbf{0}} & {{\mathbf{R}}_{{{\mathrm{B}}i{2}}} } \\ \end{array} } \right], \, {\mathbf{r}}_{{{\mathrm{B}}i{2}}} = {\mathbf{r}}_{{{\mathrm{M}}i}} + {\mathbf{R}}_{{{\mathrm{M}}i}} \cdot {\mathbf{r}}_{{{\mathrm{B}}i{2}}}^{{{\mathrm{M}}i}} {;} \hfill \\ {{\varvec{\upeta}}}_{{{\mathrm{B}}i{3}}} = \frac{1}{3}{{\varvec{\upeta}}}_{{{\mathrm{GM}},i}} , \, {{\varvec{\uprho}}}_{{{\mathrm{B}}i{3}}} = \left[ {\begin{array}{*{20}c} {{\mathbf{f}}_{{{\mathrm{B}}i3}} } \\ {{{\varvec{\uptau}}}_{{{\mathrm{B}}i3}} } \\ \end{array} } \right], \, {\mathbf{J}}_{{{\mathrm{W,B}}i3}} = \left[ {\begin{array}{*{20}c} {{\mathbf{R}}_{{{\mathrm{B}}i{3}}} } & {{\mathbf{s}}\left( {{\mathbf{r}}_{{{\mathrm{B}}i{3}}} } \right) \cdot {\mathbf{R}}_{{{\mathrm{B}}i{3}}} } \\ {\mathbf{0}} & {{\mathbf{R}}_{{{\mathrm{B}}i{3}}} } \\ \end{array} } \right], \, {\mathbf{r}}_{{{\mathrm{B}}i{3}}} = {\mathbf{r}}_{{{\mathrm{M}}i}} + {\mathbf{R}}_{{{\mathrm{M}}i}} \cdot {\mathbf{r}}_{{{\mathrm{B}}i{3}}}^{{{\mathrm{M}}i}} {;} \hfill \\ \end{gathered} $$

${{\varvec{\uprho}}}_{{{\mathrm{B}}i{1}}}$, ${{\varvec{\uprho}}}_{{{\mathrm{B}}i{2}}}$ and ${{\varvec{\uprho}}}_{{{\mathrm{B}}i{3}}}$ denote the reaction wrench applied at joints B_*i*1_, B_*i*2_ and B_*i*3_, respectively; ${{\varvec{\upeta}}}_{{{\mathrm{B}}i{1}}}$, ${{\varvec{\upeta}}}_{{{\mathrm{B}}i{2}}}$ and ${{\varvec{\upeta}}}_{{{\mathrm{B}}i{3}}}$ denote the equivalent external wrench for joints B_*i*1_, B_*i*2_ and B_*i*3_ applied at point M, respectively; ${\mathbf{s}}\left( {{\mathbf{r}}_{{{\mathrm{B}}i{1}}} } \right)$/${\mathbf{s}}\left( {{\mathbf{r}}_{{{\mathrm{B}}i{2}}} } \right)$/${\mathbf{s}}\left( {{\mathbf{r}}_{{{\mathrm{B}}i{3}}} } \right)$ represents the skew-matrix of the position vector ${\mathbf{r}}_{{{\mathrm{B}}i{1}}}$/${\mathbf{r}}_{{{\mathrm{B}}i{2}}}$/${\mathbf{r}}_{{{\mathrm{B}}i{3}}}$.

### Static equilibrium equation of joints A_1***j***_

In order to evaluate the influence of the weight of the mobile platform and links on the joint A_*ij*_, it is necessary to analysis the static force/torque balance at point A_*ij*_, as shown in Fig. 3b. The static equilibrium equations at point B_*ij*_ can be displayed as

11a $$ \left\{ {\begin{array}{*{20}l} {\frac{1}{6}m_{{{\mathrm{MP}}}} \cdot g \cdot {\mathbf{z}}{ + }m_{{i{1}}} \cdot g \cdot {\mathbf{z}} = {\mathbf{R}}_{{{\mathrm{A}}i{1}}} \cdot {\mathbf{f}}_{{{\mathrm{A}}i{1}}} } \hfill \\ \begin{gathered} \frac{1}{6}m_{{{\mathrm{MP}}}} \cdot g \cdot \left( {{\mathbf{R}}_{{\mathrm{M}}} \cdot {\mathbf{r}}_{{{\mathrm{GM}}}}^{{\mathrm{M}}} } \right) \times {\mathbf{z}} + m_{{i{1}}} \cdot g \cdot \left( {{\mathbf{R}}_{{\mathrm{M}}} \cdot {\mathbf{r}}_{{{\mathrm{M}}i}}^{{\mathrm{M}}} + {\mathbf{R}}_{{{\mathrm{M}}i}} \cdot {\mathbf{r}}_{{{\mathrm{B}}i{1}}}^{{{\mathrm{M}}i}} + {\mathbf{R}}_{{{\mathrm{B}}i1}} \cdot {\mathbf{r}}_{{{\mathrm{G}}i{1}}}^{{{\mathrm{B}}i1}} } \right) \times {\mathbf{z}} \, \hfill \\ = \left( {{\mathbf{R}}_{{\mathrm{M}}} \cdot {\mathbf{r}}_{{{\mathrm{M}}i}}^{{\mathrm{M}}} + {\mathbf{R}}_{{{\mathrm{M}}i}} \cdot {\mathbf{r}}_{{{\mathrm{B}}i{1}}}^{{{\mathrm{M}}i}} + {\mathbf{R}}_{{{\mathrm{B}}i{1}}} \cdot {\mathbf{r}}_{{{\mathrm{A}}i{1}}}^{{{\mathrm{B}}i{1}}} } \right) \times \left( {{\mathbf{R}}_{{{\mathrm{A}}i{1}}} \cdot {\mathbf{f}}_{{{\mathrm{A}}i{1}}} } \right) + {\mathbf{R}}_{{{\mathrm{A}}i{1}}} \cdot {{\varvec{\uptau}}}_{{{\mathrm{A}}i{1}}} \hfill \\ \end{gathered} \hfill \\ \end{array} } \right. $$

11b $$ \left\{ {\begin{array}{*{20}l} {\frac{1}{6}m_{{{\mathrm{MP}}}} \cdot g \cdot {\mathbf{z}}{ + }m_{{i{2}}} \cdot g \cdot {\mathbf{z}} = {\mathbf{R}}_{{{\mathrm{A}}i{2}}} \cdot {\mathbf{f}}_{{{\mathrm{A}}i{2}}} } \hfill \\ \begin{gathered} \frac{1}{6}m_{{{\mathrm{MP}}}} \cdot g \cdot \left( {{\mathbf{R}}_{{\mathrm{M}}} \cdot {\mathbf{r}}_{{{\mathrm{GM}}}}^{{\mathrm{M}}} } \right) \times {\mathbf{z}} + m_{{i{2}}} \cdot g \cdot \left( {{\mathbf{R}}_{{\mathrm{M}}} \cdot {\mathbf{r}}_{{{\mathrm{M}}i}}^{{\mathrm{M}}} + {\mathbf{R}}_{{{\mathrm{M}}i}} \cdot {\mathbf{r}}_{{{\mathrm{B}}i{2}}}^{{{\mathrm{M}}i}} + {\mathbf{R}}_{{{\mathrm{B}}i{2}}} \cdot {\mathbf{r}}_{{{\mathrm{G}}i{2}}}^{{{\mathrm{B}}i{2}}} } \right) \times {\mathbf{z}} \hfill \\ = \left( {{\mathbf{R}}_{{\mathrm{M}}} \cdot {\mathbf{r}}_{{{\mathrm{M}}i}}^{{\mathrm{M}}} + {\mathbf{R}}_{{{\mathrm{M}}i}} \cdot {\mathbf{r}}_{{{\mathrm{B}}i{2}}}^{{{\mathrm{M}}i}} + {\mathbf{R}}_{{{\mathrm{B}}i{2}}} \cdot {\mathbf{r}}_{{{\mathrm{A}}i{2}}}^{{{\mathrm{B}}i{2}}} } \right) \times \left( {{\mathbf{R}}_{{{\mathrm{A}}i{2}}} \cdot {\mathbf{f}}_{{{\mathrm{A}}i{2}}} } \right) + {\mathbf{R}}_{{{\mathrm{A}}i{2}}} \cdot {{\varvec{\uptau}}}_{{{\mathrm{A}}i{2}}} \hfill \\ \end{gathered} \hfill \\ \end{array} } \right. $$

11c $$ \left\{ {\begin{array}{*{20}l} {\frac{1}{6}m_{{{\mathrm{MP}}}} \cdot g \cdot {\mathbf{z}}{ + }m_{{i{3}}} \cdot g \cdot {\mathbf{z}} = {\mathbf{R}}_{{{\mathrm{A13}}}} \cdot {\mathbf{f}}_{{{\mathrm{A13}}}} } \hfill \\ \begin{gathered} \frac{1}{6}m_{{{\mathrm{MP}}}} \cdot g \cdot \left( {{\mathbf{R}}_{{\mathrm{M}}} \cdot {\mathbf{r}}_{{{\mathrm{GM}}}}^{{\mathrm{M}}} } \right) \times {\mathbf{z}} + m_{{i{3}}} \cdot g \cdot \left( {{\mathbf{R}}_{{\mathrm{M}}} \cdot {\mathbf{r}}_{{{\mathrm{M}}i}}^{{\mathrm{M}}} + {\mathbf{R}}_{{{\mathrm{M}}i}} \cdot {\mathbf{r}}_{{{\mathrm{B}}i{3}}}^{{{\mathrm{M}}i}} + {\mathbf{R}}_{{{\mathrm{B}}i{3}}} \cdot {\mathbf{r}}_{{{\mathrm{G}}i{3}}}^{{{\mathrm{B}}i{3}}} } \right) \times {\mathbf{z}} \hfill \\ = \left( {{\mathbf{R}}_{{\mathrm{M}}} \cdot {\mathbf{r}}_{{{\mathrm{M}}i}}^{{\mathrm{M}}} + {\mathbf{R}}_{{{\mathrm{M}}i}} \cdot {\mathbf{r}}_{{{\mathrm{B}}i{3}}}^{{{\mathrm{M}}i}} + {\mathbf{R}}_{{{\mathrm{B}}i{3}}} \cdot {\mathbf{r}}_{{{\mathrm{A}}i{3}}}^{{{\mathrm{B}}i{3}}} } \right) \times \left( {{\mathbf{R}}_{{{\mathrm{A}}i{3}}} \cdot {\mathbf{f}}_{{{\mathrm{A}}i{3}}} } \right) + {\mathbf{R}}_{{{\mathrm{A}}i{3}}} \cdot {{\varvec{\uptau}}}_{{{\mathrm{A}}i{3}}} \hfill \\ \end{gathered} \hfill \\ \end{array} } \right. $$

where

$$ {\mathbf{f}}_{{{\mathrm{A}}i{1}}} = \left[ {\begin{array}{*{20}c} {f_{{{\mathrm{A}}i{1,}x}} } & {f_{{{\mathrm{A}}i{1,}y}} } & {f_{{{\mathrm{A}}i{1,}z}} } \\ \end{array} } \right]^{{\mathrm{T}}} , \, {{\varvec{\uptau}}}_{{{\mathrm{A}}i{1}}} = \left[ {\begin{array}{*{20}c} {\tau_{{{\mathrm{A}}i{1,}x}} } & {\tau_{{{\mathrm{A}}i{1,}y}} } & {\tau_{{{\mathrm{A}}i{1,}z}} } \\ \end{array} } \right]^{{\mathrm{T}}} ; $$

$$ {\mathbf{f}}_{{{\mathrm{A}}i{2}}} = \left[ {\begin{array}{*{20}c} {f_{{{\mathrm{A}}i{2,}x}} } & {f_{{{\mathrm{A}}i{2,}y}} } & {f_{{{\mathrm{A}}i{2,}z}} } \\ \end{array} } \right]^{{\mathrm{T}}} , \, {{\varvec{\uptau}}}_{{{\mathrm{A}}i{2}}} = \left[ {\begin{array}{*{20}c} {\tau_{{{\mathrm{A}}i{2,}x}} } & {\tau_{{{\mathrm{A}}i{2,}y}} } & {\tau_{{{\mathrm{A}}i{2,}z}} } \\ \end{array} } \right]^{{\mathrm{T}}} ; $$

$$ {\mathbf{f}}_{{{\mathrm{A}}i{3}}} = \left[ {\begin{array}{*{20}c} {f_{{{\mathrm{A}}i{3,}x}} } & {f_{{{\mathrm{A}}i{3,}y}} } & {f_{{{\mathrm{A}}i{3,}z}} } \\ \end{array} } \right]^{{\mathrm{T}}} , \, {{\varvec{\uptau}}}_{{{\mathrm{A}}i{3}}} = \left[ {\begin{array}{*{20}c} {\tau_{{{\mathrm{A}}i{3,}x}} } & {\tau_{{{\mathrm{A}}i{3,}y}} } & {\tau_{{{\mathrm{A}}i{3,}z}} } \\ \end{array} } \right]^{{\mathrm{T}}} ; $$

*m*_*i*1_, *m*_*i*2_ and *m*_*i*3_ are the mass of links L_*i*1_, L_*i*2_ and L_*i*3_, respectively; G_*i*1_, G_*i*2_ and G_*i*3_ are the mass center of links L_*i*1_, L_*i*2_ and L_*i*3_, respectively; ${\mathbf{f}}_{{{\mathrm{A}}i{1}}}$ and ${{\varvec{\uptau}}}_{{{\mathrm{A}}i{1}}}$ denote the reaction force and torque applied at point A_*i*1_, ${\mathbf{f}}_{{{\mathrm{A}}i{2}}}$ and ${{\varvec{\uptau}}}_{{{\mathrm{A}}i{2}}}$ denote the reaction force and torque applied at point A_*i*2_, ${\mathbf{f}}_{{{\mathrm{A}}i{3}}}$ and ${{\varvec{\uptau}}}_{{{\mathrm{A}}i{3}}}$ denote the reaction force and torque applied at point A_*i*3_; ${\mathbf{r}}_{{{\mathrm{G}}i{1}}}^{{{\mathrm{B}}i1}}$ denotes the position vector from point B_*i*1_ to point G_*i*1_ expressed in the coordinate system {B_*i*1_}, ${\mathbf{r}}_{{{\mathrm{G}}i{2}}}^{{{\mathrm{B}}i{2}}}$ denotes the position vector from point B_*i*2_ to point G_*i*2_ expressed in the coordinate system {B_*i*2_}, ${\mathbf{r}}_{{{\mathrm{G}}i{3}}}^{{{\mathrm{B}}i{3}}}$ denotes the position vector from point B_*i*3_ to point G_*i*3_ expressed in the coordinate system {B_*i*3_}; ${\mathbf{r}}_{{{\mathrm{A}}i{1}}}^{{{\mathrm{B}}i{1}}}$ denotes the position vector from point B_*i*1_ to point A_*i*1_ expressed in the coordinate system {B_*i*1_}, ${\mathbf{r}}_{{{\mathrm{A}}i{2}}}^{{{\mathrm{B}}i{2}}}$ denotes the position vector from point B_*i*2_ to point A_*i*2_ expressed in the coordinate system {B_*i*2_}, ${\mathbf{r}}_{{{\mathrm{A}}i{3}}}^{{{\mathrm{B}}i{3}}}$ denotes the position vector from point B_*i*3_ to point A_*i*3_ expressed in the coordinate system {B_*i*3_}.

According to the decoupling of Eq. (11), the reaction force acting on the joints A_*i*1_, A_*i*2_ and A_*i*3_ can be obtained

12 $$ {{\varvec{\uprho}}}_{{{\mathrm{A}}i{1}}} { = }{\mathbf{J}}_{{{\mathrm{W,A}}i{1}}}^{{\mathrm{T}}} \cdot {{\varvec{\upeta}}}_{{{\mathrm{A}}i{1}}} , \, {{\varvec{\uprho}}}_{{{\mathrm{A}}i{2}}} { = }{\mathbf{J}}_{{{\mathrm{W,A}}i2}}^{{\mathrm{T}}} \cdot {{\varvec{\upeta}}}_{{{\mathrm{A}}i{2}}} , \, {{\varvec{\uprho}}}_{{{\mathrm{A}}i{3}}} { = }{\mathbf{J}}_{{{\mathrm{W,A}}i3}}^{{\mathrm{T}}} \cdot {{\varvec{\upeta}}}_{{{\mathrm{A}}i{3}}} $$

where

$$ \begin{gathered} {{\varvec{\upeta}}}_{{{\mathrm{A}}i{1}}} = {{\varvec{\upeta}}}_{{i{1}}} + \frac{1}{3}{{\varvec{\upeta}}}_{{{\mathrm{GM}},i}} , \, {{\varvec{\upeta}}}_{{i{1}}} = m_{{i{1}}} \cdot g \cdot \left[ {\begin{array}{*{20}c} {\mathbf{z}} \\ {\left( {{\mathbf{r}}_{{{\mathrm{B}}i1}} + {\mathbf{R}}_{{{\mathrm{B}}i{1}}} \cdot {\mathbf{r}}_{{{\mathrm{G}}i{1}}}^{{{\mathrm{B}}i{1}}} } \right) \times {\mathbf{z}}} \\ \end{array} } \right], \, {{\varvec{\uprho}}}_{{{\mathrm{A}}i{1}}} = \left[ {\begin{array}{*{20}c} {{\mathbf{f}}_{{{\mathrm{A}}i1}} } \\ {{{\varvec{\uptau}}}_{{{\mathrm{A}}i1}} } \\ \end{array} } \right], \hfill \\ {\mathbf{J}}_{{{\mathrm{W,A}}i1}} = \left[ {\begin{array}{*{20}c} {{\mathbf{R}}_{{{\mathrm{A}}i{1}}} } & {{\mathbf{s}}\left( {{\mathbf{r}}_{{{\mathrm{A}}i{1}}} } \right) \cdot {\mathbf{R}}_{{{\mathrm{A}}i{1}}} } \\ {\mathbf{0}} & {{\mathbf{R}}_{{{\mathrm{A}}i{1}}} } \\ \end{array} } \right], \, {\mathbf{r}}_{{{\mathrm{A}}i{1}}} = {\mathbf{r}}_{{{\mathrm{B}}i1}} + {\mathbf{R}}_{{{\mathrm{B}}i{1}}} \cdot {\mathbf{r}}_{{{\mathrm{A}}i{1}}}^{{{\mathrm{B}}i{1}}} {;} \hfill \\ \end{gathered} $$

$$ \begin{gathered} {{\varvec{\upeta}}}_{{{\mathrm{A}}i{2}}} = {{\varvec{\upeta}}}_{{i{2}}} + \frac{1}{3}{{\varvec{\upeta}}}_{{{\mathrm{GM}},i}} , \, {{\varvec{\upeta}}}_{{i{2}}} = m_{{i{2}}} \cdot g \cdot \left[ {\begin{array}{*{20}c} {\mathbf{z}} \\ {\left( {{\mathbf{r}}_{{{\mathrm{B}}i{2}}} + {\mathbf{R}}_{{{\mathrm{B}}i{2}}} \cdot {\mathbf{r}}_{{{\mathrm{G}}i{2}}}^{{{\mathrm{B}}i{2}}} } \right) \times {\mathbf{z}}} \\ \end{array} } \right], \, {{\varvec{\uprho}}}_{{{\mathrm{A}}i{2}}} = \left[ {\begin{array}{*{20}c} {{\mathbf{f}}_{{{\mathrm{A}}i2}} } \\ {{{\varvec{\uptau}}}_{{{\mathrm{A}}i2}} } \\ \end{array} } \right], \hfill \\ {\mathbf{J}}_{{{\mathrm{W,A}}i2}} = \left[ {\begin{array}{*{20}c} {{\mathbf{R}}_{{{\mathrm{A}}i{2}}} } & {{\mathbf{s}}\left( {{\mathbf{r}}_{{{\mathrm{A}}i{2}}} } \right) \cdot {\mathbf{R}}_{{{\mathrm{A}}i{2}}} } \\ {\mathbf{0}} & {{\mathbf{R}}_{{{\mathrm{A}}i{2}}} } \\ \end{array} } \right], \, {\mathbf{r}}_{{{\mathrm{A}}i{2}}} = {\mathbf{r}}_{{{\mathrm{B}}i{2}}} + {\mathbf{R}}_{{{\mathrm{B}}i{2}}} \cdot {\mathbf{r}}_{{{\mathrm{A}}i{2}}}^{{{\mathrm{B}}i{2}}} {;} \hfill \\ \end{gathered} $$

$$ \begin{gathered} {{\varvec{\upeta}}}_{{{\mathrm{A}}i{3}}} = {{\varvec{\upeta}}}_{{i{3}}} + \frac{1}{3}{{\varvec{\upeta}}}_{{{\mathrm{GM}},i}} , \, {{\varvec{\upeta}}}_{{i{3}}} = m_{{i{3}}} \cdot g \cdot \left[ {\begin{array}{*{20}c} {\mathbf{z}} \\ {\left( {{\mathbf{r}}_{{{\mathrm{B}}i{3}}} + {\mathbf{R}}_{{{\mathrm{B}}i{3}}} \cdot {\mathbf{r}}_{{{\mathrm{G}}i{3}}}^{{{\mathrm{B}}i{3}}} } \right) \times {\mathbf{z}}} \\ \end{array} } \right], \, {{\varvec{\uprho}}}_{{{\mathrm{A}}i{3}}} = \left[ {\begin{array}{*{20}c} {{\mathbf{f}}_{{{\mathrm{A}}i3}} } \\ {{{\varvec{\uptau}}}_{{{\mathrm{A}}i3}} } \\ \end{array} } \right], \hfill \\ {\mathbf{J}}_{{{\mathrm{W,A}}i3}} = \left[ {\begin{array}{*{20}c} {{\mathbf{R}}_{{{\mathrm{A}}i{3}}} } & {{\mathbf{s}}\left( {{\mathbf{r}}_{{{\mathrm{A}}i{3}}} } \right) \cdot {\mathbf{R}}_{{{\mathrm{A}}i{3}}} } \\ {\mathbf{0}} & {{\mathbf{R}}_{{{\mathrm{A}}i{3}}} } \\ \end{array} } \right], \, {\mathbf{r}}_{{{\mathrm{A}}i{3}}} = {\mathbf{r}}_{{{\mathrm{B}}i{3}}} + {\mathbf{R}}_{{{\mathrm{B}}i{3}}} \cdot {\mathbf{r}}_{{{\mathrm{A}}i{3}}}^{{{\mathrm{B}}i{3}}} {;} \hfill \\ \end{gathered} $$

${{\varvec{\upeta}}}_{{{\mathrm{A}}i{1}}}$, ${{\varvec{\upeta}}}_{{{\mathrm{A}}i{2}}}$ and ${{\varvec{\upeta}}}_{{{\mathrm{A}}i{3}}}$ denote the resultant external wrench for joint A_*i*1_, A_*i*2_ and A_*i*3_ applied at point M, respectively; ${{\varvec{\upeta}}}_{{i{1}}}$, ${{\varvec{\upeta}}}_{{i{2}}}$ and ${{\varvec{\upeta}}}_{{i{3}}}$ denote the equivalent gravitational wrench of links L_*i*1_, L_*i*2_ and L_*i*3_ applied at point M, respectively; ${{\varvec{\uprho}}}_{{{\mathrm{A}}i{1}}}$, ${{\varvec{\uprho}}}_{{{\mathrm{A}}i{2}}}$ and ${{\varvec{\uprho}}}_{{{\mathrm{A}}i{3}}}$ denote the reaction wrench applied at joints A_*i*1_, A_*i*2_ and A_*i*3_, respectively; ${\mathbf{s}}\left( {{\mathbf{r}}_{{{\mathrm{A}}i{1}}} } \right)$/${\mathbf{s}}\left( {{\mathbf{r}}_{{{\mathrm{A}}i{2}}} } \right)$/${\mathbf{s}}\left( {{\mathbf{r}}_{{{\mathrm{A}}i{3}}} } \right)$ represents the skew-matrix of the position vector ${\mathbf{r}}_{{{\mathrm{A}}i{1}}}$/${\mathbf{r}}_{{{\mathrm{A}}i{2}}}$/${\mathbf{r}}_{{{\mathrm{A}}i{3}}}$.

### Static equilibrium equation of joint A_***i***_

In order to evaluate the influence of the weight of the mobile platform and links on the joint A_*i*_, it is necessary to analysis the static force/torque balance at point A_*i*_, as shown in Fig. 3b. The resultant static equilibrium equations at point A_*ij*_ can be displayed as

13 $$ \left\{ {\begin{array}{*{20}l} {\frac{1}{2}m_{{{\mathrm{MP}}}} \cdot g \cdot {\mathbf{z}}{ + }\left( {m_{{i{1}}} + m_{{i{2}}} + m_{{i{3}}} } \right) \cdot g \cdot {\mathbf{z}} = {\mathbf{R}}_{{{\mathrm{A}}i}} \cdot {\mathbf{f}}_{{{\mathrm{A}}i}} } \hfill \\ \begin{gathered} \frac{1}{2}m_{{{\mathrm{MP}}}} \cdot g \cdot \left( {{\mathbf{R}}_{{\mathrm{M}}} \cdot {\mathbf{r}}_{{{\mathrm{GM}}}}^{{\mathrm{M}}} } \right) \times {\mathbf{z}} + m_{{i{1}}} \cdot g \cdot \left( {{\mathbf{R}}_{{\mathrm{M}}} \cdot {\mathbf{r}}_{{{\mathrm{M}}i}}^{{\mathrm{M}}} + {\mathbf{R}}_{{{\mathrm{M}}i}} \cdot {\mathbf{r}}_{{{\mathrm{B}}i{1}}}^{{{\mathrm{M}}i}} + {\mathbf{R}}_{{{\mathrm{B}}i{1}}} \cdot {\mathbf{r}}_{{{\mathrm{G}}i{1}}}^{{{\mathrm{B}}i{1}}} } \right) \times {\mathbf{z}} \hfill \\ + m_{{i{2}}} \cdot g \cdot \left( {{\mathbf{R}}_{{\mathrm{M}}} \cdot {\mathbf{r}}_{{{\mathrm{M}}i}}^{{\mathrm{M}}} + {\mathbf{R}}_{{{\mathrm{M}}i}} \cdot {\mathbf{r}}_{{{\mathrm{B}}i{2}}}^{{{\mathrm{M}}i}} + {\mathbf{R}}_{{{\mathrm{B}}i{2}}} \cdot {\mathbf{r}}_{{{\mathrm{G}}i{2}}}^{{{\mathrm{B}}i{2}}} } \right) \times {\mathbf{z}} \hfill \\ + m_{{i{3}}} \cdot g \cdot \left( {{\mathbf{R}}_{{\mathrm{M}}} \cdot {\mathbf{r}}_{{{\mathrm{M}}i}}^{{\mathrm{M}}} + {\mathbf{R}}_{{{\mathrm{M}}i}} \cdot {\mathbf{r}}_{{{\mathrm{B}}i{3}}}^{{{\mathrm{M}}i}} + {\mathbf{R}}_{{{\mathrm{B}}i{3}}} \cdot {\mathbf{r}}_{{{\mathrm{G}}i{3}}}^{{{\mathrm{B}}i{3}}} } \right) \times {\mathbf{z}} \hfill \\ = \left( {{\mathbf{R}}_{{\mathrm{M}}} \cdot {\mathbf{r}}_{{{\mathrm{M}}i}}^{{\mathrm{M}}} + {\mathbf{R}}_{{{\mathrm{M}}i}} \cdot {\mathbf{r}}_{{{\mathrm{A}}i}}^{{{\mathrm{M}}i}} } \right) \times \left( {{\mathbf{R}}_{{{\mathrm{A}}i}} \cdot {\mathbf{f}}_{{{\mathrm{A}}i}} } \right) + {\mathbf{R}}_{{{\mathrm{A}}i}} \cdot {{\varvec{\uptau}}}_{{{\mathrm{A}}i}} \hfill \\ \end{gathered} \hfill \\ \end{array} } \right. $$

where

$$ {\mathbf{f}}_{{{\mathrm{A}}i}} = \left[ {\begin{array}{*{20}c} {f_{{{\mathrm{A}}i{,}x}} } & {f_{{{\mathrm{A}}i{,}y}} } & {f_{{{\mathrm{A}}i{,}z}} } \\ \end{array} } \right]^{{\mathrm{T}}} , \, {{\varvec{\uptau}}}_{{{\mathrm{A}}i}} = \left[ {\begin{array}{*{20}c} {\tau_{{{\mathrm{A}}i{,}x}} } & {\tau_{{{\mathrm{A}}i{,}y}} } & {\tau_{{{\mathrm{A}}i{,}z}} } \\ \end{array} } \right]^{{\mathrm{T}}} ; $$

${\mathbf{f}}_{{{\mathrm{A}}i}}$ and ${{\varvec{\uptau}}}_{{{\mathrm{A}}i}}$ denote the reaction force and torque applied at point A_*i*_, ${\mathbf{r}}_{{{\mathrm{A}}i}}^{{{\mathrm{M}}i}}$ denotes the position vector from point M_*i*_ to point A_*i*_ expressed in the coordinate system {M_*i*_}.

According to the decoupling of Eq. (13), the reaction force acting on the joint A_*i*_ can be obtained

14 $$ {{\varvec{\uprho}}}_{{{\mathrm{A}}i}} { = }{\mathbf{J}}_{{{\mathrm{W,A}}i}}^{{\mathrm{T}}} \cdot {{\varvec{\upeta}}}_{{{\mathrm{A}}i}} $$

where

$$ {{\varvec{\upeta}}}_{{{\mathrm{A}}i}} = {{\varvec{\upeta}}}_{{{\mathrm{GM}},i}} + {{\varvec{\upeta}}}_{{i{1}}} + {{\varvec{\upeta}}}_{{i{2}}} + {{\varvec{\upeta}}}_{{i{3}}} , \, {{\varvec{\uprho}}}_{{{\mathrm{A}}i}} = \left[ {\begin{array}{*{20}c} {{\mathbf{f}}_{{{\mathrm{A}}i}} } \\ {{{\varvec{\uptau}}}_{{{\mathrm{A}}i}} } \\ \end{array} } \right], \, {\mathbf{J}}_{{{\mathrm{W,A}}i}} = \left[ {\begin{array}{*{20}c} {{\mathbf{R}}_{{{\mathrm{A}}i}} } & {{\mathbf{s}}\left( {{\mathbf{r}}_{{{\mathrm{A}}i}} } \right) \cdot {\mathbf{R}}_{{{\mathrm{A}}i}} } \\ {\mathbf{0}} & {{\mathbf{R}}_{{{\mathrm{A}}i}} } \\ \end{array} } \right], \, {\mathbf{r}}_{{{\mathrm{A}}i}} = {\mathbf{r}}_{{{\mathrm{M}}i}} + {\mathbf{R}}_{{{\mathrm{M}}i}} \cdot {\mathbf{r}}_{{{\mathrm{A}}i}}^{{{\mathrm{M}}i}} {;} $$

${{\varvec{\upeta}}}_{{{\mathrm{A}}i}}$ denotes the resultant external wrench for joint A_*i*_ applied at point M, ${{\varvec{\uprho}}}_{{{\mathrm{A}}i}}$ denotes the reaction wrench applied at joint A_*i*_, ${\mathbf{s}}\left( {{\mathbf{r}}_{{{\mathrm{A}}i}} } \right)$ represents the skew-matrix of the position vector ${\mathbf{r}}_{{{\mathrm{A}}i}}$.

## Kinematic analysis of the Biglide parallel robot

In this section, the kinematic analysis of the Biglide parallel robot is carried out so that the Jacobian matrix of each substructure is derived^27–33^. Due to the position relationship and series/parallel relationship of each substructure, the kinematic equilibrium equation is established for each substructure of the Biglide parallel robot.

### Kinematic modeling for 3-RR parallelogram-link structure

In order to obtain the Jacobian matrices of each kinematic chain in the 3-RR, it is necessary to analyze the kinematic transitions from point A_*i*_ to B_*i*_ through each chain, as shown in Fig. 3b. The kinematic homogenous matrix of the first chain in the left 3-RR Pl can be expressed as

15 $$ {\mathbf{T}}_{{{\mathrm{Pl}},1}}^{1} = {\mathbf{T}}_{{{\mathrm{Bl}},11}}^{1} \cdot {\mathbf{T}}_{{{\mathrm{Ps}}}} \left( {q_{{{\mathrm{Pl}},11}}^{1} } \right) \cdot {\mathbf{T}}_{{{\mathrm{link}},1}}^{1} \cdot {\mathbf{T}}_{{{\mathrm{Vs}}}} \left( {\theta_{{{\mathrm{Pl}},11}}^{1} ,...,\theta_{{{\mathrm{Pl}},16}}^{1} } \right) \cdot {\mathbf{T}}_{{{\mathrm{Ps}}}} \left( {q_{{{\mathrm{Pl}},12}}^{1} } \right) \cdot {\mathbf{T}}_{{{\mathrm{Bl}},12}}^{1} $$

where

$$ {\mathbf{T}}_{{{\mathrm{Bl}},11}}^{1} = \left[ {\begin{array}{*{20}c} {{\mathbf{R}}\left( {y, - q} \right)} & {\mathbf{0}} \\ {\mathbf{0}} & 1 \\ \end{array} } \right] \cdot \left[ {\begin{array}{*{20}c} {{\mathbf{I}}_{3} } & {{\mathbf{P}}\left( {x, - h} \right)} \\ {\mathbf{0}} & 1 \\ \end{array} } \right], \, {\mathbf{T}}_{{{\mathrm{Ps}}}} \left( {q_{{{\mathrm{Pl}},11}}^{1} } \right) = \left[ {\begin{array}{*{20}c} {{\mathbf{R}}\left( {y,q} \right)} & {\mathbf{0}} \\ {\mathbf{0}} & 1 \\ \end{array} } \right] \cdot \left[ {\begin{array}{*{20}c} {{\mathbf{R}}\left( {y,q_{{{\mathrm{Pl}},11}}^{1} } \right)} & {\mathbf{0}} \\ {\mathbf{0}} & 1 \\ \end{array} } \right], $$

$$ \begin{gathered} {\mathbf{T}}_{{{\mathrm{link}},1}}^{1} = \left[ {\begin{array}{*{20}c} {{\mathbf{I}}_{3} } & {{\mathbf{P}}\left( {x,L} \right)} \\ {\mathbf{0}} & 1 \\ \end{array} } \right], \, {\mathbf{T}}_{{{\mathrm{Vs}}}} \left( {\theta_{{{\mathrm{Pl}},11}}^{1} ,...,\theta_{{{\mathrm{Pl}},16}}^{1} } \right) = \left[ {\begin{array}{*{20}c} {{\mathbf{I}}_{3} } & {{\mathbf{P}}\left( {x,\theta_{{{\mathrm{Pl}},11}}^{1} } \right)} \\ {\mathbf{0}} & 1 \\ \end{array} } \right] \cdot \left[ {\begin{array}{*{20}c} {{\mathbf{I}}_{3} } & {{\mathbf{P}}\left( {y,\theta_{{{\mathrm{Pl}},12}}^{1} } \right)} \\ {\mathbf{0}} & 1 \\ \end{array} } \right] \hfill \\ \cdot \left[ {\begin{array}{*{20}c} {{\mathbf{I}}_{3} } & {{\mathbf{P}}\left( {z,\theta_{{{\mathrm{Pl}},13}}^{1} } \right)} \\ {\mathbf{0}} & 1 \\ \end{array} } \right] \cdot \left[ {\begin{array}{*{20}c} {{\mathbf{R}}\left( {x,\theta_{{{\mathrm{Pl}},14}}^{1} } \right)} & {\mathbf{0}} \\ {\mathbf{0}} & 1 \\ \end{array} } \right] \cdot \left[ {\begin{array}{*{20}c} {{\mathbf{R}}\left( {y,\theta_{{{\mathrm{Pl}},15}}^{1} } \right)} & {\mathbf{0}} \\ {\mathbf{0}} & 1 \\ \end{array} } \right] \cdot \left[ {\begin{array}{*{20}c} {{\mathbf{R}}\left( {z,\theta_{{{\mathrm{Pl}},16}}^{1} } \right)} & {\mathbf{0}} \\ {\mathbf{0}} & 1 \\ \end{array} } \right], \hfill \\ \end{gathered} $$

$$ {\mathbf{T}}_{{{\mathrm{Ps}}}} \left( {q_{{{\mathrm{Pl}},12}}^{1} } \right) = \left[ {\begin{array}{*{20}c} {{\mathbf{R}}\left( {y, - q} \right)} & {\mathbf{0}} \\ {\mathbf{0}} & 1 \\ \end{array} } \right] \cdot \left[ {\begin{array}{*{20}c} {{\mathbf{R}}\left( {y,q_{{{\mathrm{Pl}},12}}^{1} } \right)} & {\mathbf{0}} \\ {\mathbf{0}} & 1 \\ \end{array} } \right], \, {\mathbf{T}}_{{{\mathrm{Bl}},12}}^{1} = \left[ {\begin{array}{*{20}c} {{\mathbf{I}}_{3} } & {{\mathbf{P}}\left( {x,h} \right)} \\ {\mathbf{0}} & 1 \\ \end{array} } \right] \cdot \left[ {\begin{array}{*{20}c} {{\mathbf{R}}\left( {y,q} \right)} & {\mathbf{0}} \\ {\mathbf{0}} & 1 \\ \end{array} } \right], $$

P(.) represents a 3 × 1 displacement vector that translates the corresponding distance along the corresponding axis, **I**_3_ is a 3 × 3 identity matrix.

The position and direction of the end point B1 can be extracted from the matrix ${\mathbf{T}}_{{{\mathrm{Pl}},1}}^{1}$ in a standard way^27,33,34^, whereby the kinematic model can be rewritten in the form of a vector function as follows

16 $$ {\mathbf{t}}_{{{\mathrm{Pl}},1}}^{1} = {\mathbf{g}}\left( {{{\varvec{\uptheta}}}_{{{\mathrm{Pl}},1}}^{1} ,{\mathbf{q}}_{{{\mathrm{Pl}},1}}^{1} } \right) $$

where ${\mathbf{t}}_{{{\mathrm{Pl}},1}}^{1}$ is a vector that describes the position and direction of the end point B_1_, ${{\varvec{\uptheta}}}_{{{\mathrm{Pl}},1}}^{1}$ is a vector collecting all virtual spring coordinates, ${\mathbf{q}}_{{{\mathrm{Pl}},1}}^{1}$ is a vector containing all passive joint coordinates.

According to the principle of virtual work, the virtual displacement $\delta {\mathbf{t}}_{{{\mathrm{Pl}},1}}^{1}$ can be computed from the linearized geometrical model:

17 $$ \delta {\mathbf{t}}_{{{\mathrm{Pl}},1}}^{1} = {\mathbf{J}}_{{{\uptheta },{\mathrm{Pl}},1}}^{1} \cdot \delta {{\varvec{\uptheta}}}_{{{\mathrm{Pl}},1}}^{1} + {\mathbf{J}}_{{{\mathrm{q}},{\mathrm{Pl}},1}}^{1} \cdot \delta {\mathbf{q}}_{{{\mathrm{Pl}},1}}^{1} $$

which includes the Jacobian matrices:

18 $$ \left\{ {\begin{array}{*{20}l} {{\mathbf{J}}_{{{\uptheta },{\mathrm{Pl}},1}}^{1} = \frac{\partial }{{\partial {{\varvec{\uptheta}}}_{{{\mathrm{Pl}},1}}^{1} }}{\mathbf{g}}\left( {{{\varvec{\uptheta}}}_{{{\mathrm{Pl}},1}}^{1} ,{\mathbf{q}}_{{{\mathrm{Pl}},1}}^{1} } \right) = \left[ {{\mathbf{J}}_{{{\uptheta },{\mathrm{Pl}},11}}^{1} , \ldots ,{\mathbf{J}}_{{{\uptheta },{\mathrm{Pl}},16}}^{1} } \right]} \hfill \\ {{\mathbf{J}}_{{{\mathrm{q}},{\mathrm{Pl}},1}}^{1} = \frac{\partial }{{\partial {\mathbf{q}}_{{{\mathrm{Pl}},1}}^{1} }}{\mathbf{g}}\left( {{{\varvec{\uptheta}}}_{{{\mathrm{Pl}},1}}^{1} ,{\mathbf{q}}_{{{\mathrm{Pl}},1}}^{1} } \right) = \left[ {{\mathbf{J}}_{{{\mathrm{q}},{\mathrm{Pl}},11}}^{1} ,{\mathbf{J}}_{{{\mathrm{q}},{\mathrm{Pl}},12}}^{1} } \right]} \hfill \\ \end{array} } \right. $$

where

$$\frac{\partial }{{\partial \uptheta _{{{\mathrm{Pl}},1n}}^{1} }}{\mathbf{T}}_{{{\mathrm{Pl}},1}}^{1}  = \left[ {\begin{array}{*{20}c}    {{\mathbf{s}}\left( {{\mathbf{\omega }}_{{{{\uptheta , \mathrm{Pl}}},1n}}^{1} } \right)} & {{\mathbf{p}}_{{{{\uptheta ,\mathrm{Pl}}},1n}}^{1} }  \\    {\mathbf{0}} & 0  \\   \end{array} } \right], \;{\mathbf{J}}_{{{{\uptheta }},{\mathrm{Pl}},1n}}^{1}  = \frac{\partial }{{\partial \uptheta _{{{\mathrm{Pl}},1n}}^{1} }}{\mathbf{g}}\left( {{\mathbf{\uptheta }}_{{{\mathrm{Pl}},1}}^{1} ,{\mathbf{q}}_{{{\mathrm{Pl}},1}}^{1} } \right)  = \left[ {\begin{array}{*{20}c}    {{\mathbf{p}}_{{{{\uptheta , \mathrm{Pl}}},1n}}^{1} }  \\    {{\mathbf{\omega }}_{{{{\uptheta , \mathrm{Pl}}},1n}}^{1} }  \\   \end{array} } \right];$$

$$ \frac{\partial }{{\partial q_{{{\mathrm{Pl}},1k}}^{1} }}{\mathbf{T}}_{{{\mathrm{Pl}},1}}^{1} = \left[ {\begin{array}{*{20}c} {{\mathbf{s}}\left( {{{\varvec{\upomega}}}_{{{\mathrm{q,Pl}},1k}}^{1} } \right)} & {{\mathbf{p}}_{{{\mathrm{q,Pl}},1k}}^{1} } \\ {\mathbf{0}} & 0 \\ \end{array} } \right], \, {\mathbf{J}}_{{{\mathrm{q}},{\mathrm{Pl}},1k}}^{1} = \frac{\partial }{{\partial q_{{{\mathrm{Pl}},1k}}^{1} }}{\mathbf{g}}\left( {{{\varvec{\uptheta}}}_{{{\mathrm{Pl}},1}}^{1} ,{\mathbf{q}}_{{{\mathrm{Pl}},1}}^{1} } \right) = \left[ {\begin{array}{*{20}c} {{\mathbf{p}}_{{{\mathrm{q,Pl}},1k}}^{1} } \\ {{{\varvec{\upomega}}}_{{{\mathrm{q,Pl}},1k}}^{1} } \\ \end{array} } \right]{;} $$

${\mathbf{p}}_{{{\uptheta ,{\mathrm Pl}},1n}}^{1}$ and ${\mathbf{p}}_{{{\mathrm{q,Pl}},1k}}^{1}$ denote the position vector associated with $\theta_{{{\mathrm{Pl}},1n}}^{1}{^{\prime}}$ and $q_{{{\mathrm{Pl}},1k}}^{1}{^{\prime}}$ respectively, ${\mathbf{s}}\left( {{{\varvec{\upomega}}}_{{{\uptheta ,{\mathrm Pl}},1n}}^{1} } \right)$/${\mathbf{s}}\left( {{{\varvec{\upomega}}}_{{{\mathrm{q,Pl}},1k}}^{1} } \right)$ is represents the skew-matrix of the orientation vector ${{\varvec{\upomega}}}_{{{\uptheta ,{\mathrm Pl}},1n}}^{1}$/${{\varvec{\upomega}}}_{{{\mathrm{q,Pl}},1k}}^{1}$.

According to the above theory, the Jacobian matrices of the first chain in the left 3-RR Pl can be obtained by deriving Eq. (15) as follows:

19 $$ {\mathbf{J}}_{{{\uptheta },{\mathrm{Pl}},1}}^{1} = \left[ {\begin{array}{*{20}c} 1 & 0 & 0 & 0 & {h \cdot {\mathrm{S}}q} & 0 \\ 0 & 1 & 0 & { - h \cdot {\mathrm{S}}q} & 0 & {h \cdot {\mathrm{C}}q} \\ 0 & 0 & 1 & 0 & { - h \cdot {\mathrm{C}}q} & 0 \\ 0 & 0 & 0 & 1 & 0 & 0 \\ 0 & 0 & 0 & 0 & 1 & 0 \\ 0 & 0 & 0 & 0 & 0 & 1 \\ \end{array} } \right],\;{\mathbf{J}}_{{{\mathrm{q}},{\mathrm{Pl}},1}}^{1} = \left[ {\begin{array}{*{20}c} {h \cdot {\mathrm{S}}q} & {h \cdot {\mathrm{S}}q} \\ 0 & 0 \\ { - h \cdot {\mathrm{C}}q - L} & { - h \cdot {\mathrm{C}}q} \\ 0 & 0 \\ 1 & 1 \\ 0 & 0 \\ \end{array} } \right] $$

where S_*q*_ and C_*q*_ represent sine and cosine of the angle *q*_1_, respectively.

Similarly, the Jacobian matrices for the second and third chains in the left 3-RR Pl, respectively, can be expressed as

20a $$ {\mathbf{J}}_{{{\uptheta },{\mathrm{Pl}},2}}^{1} = \left[ {\begin{array}{*{20}c} 1 & 0 & 0 & 0 & { - h \cdot {\mathrm{S}}q} & d \\ 0 & 1 & 0 & {h \cdot {\mathrm{S}}q} & 0 & { - h \cdot {\mathrm{C}}q} \\ 0 & 0 & 1 & { - d} & {h \cdot {\mathrm{C}}q} & 0 \\ 0 & 0 & 0 & 1 & 0 & 0 \\ 0 & 0 & 0 & 0 & 1 & 0 \\ 0 & 0 & 0 & 0 & 0 & 1 \\ \end{array} } \right],{\mathbf{J}}_{{{\mathrm{q}},{\mathrm{Pl}},2}}^{1} = \left[ {\begin{array}{*{20}c} { - h \cdot {\mathrm{S}}q} & { - h \cdot {\mathrm{S}}q} \\ 0 & 0 \\ {h \cdot {\mathrm{C}}q - L} & {h \cdot {\mathrm{C}}q} \\ 0 & 0 \\ 1 & 1 \\ 0 & 0 \\ \end{array} } \right]{;} $$

20b $$ {\mathbf{J}}_{{{\uptheta },{\mathrm{Pl}},3}}^{1} = \left[ {\begin{array}{*{20}c} 1 & 0 & 0 & 0 & { - h \cdot {\mathrm{S}}q} & { - d} \\ 0 & 1 & 0 & {h \cdot {\mathrm{S}}q} & 0 & { - h \cdot {\mathrm{C}}q} \\ 0 & 0 & 1 & d & {h \cdot {\mathrm{C}}q} & 0 \\ 0 & 0 & 0 & 1 & 0 & 0 \\ 0 & 0 & 0 & 0 & 1 & 0 \\ 0 & 0 & 0 & 0 & 0 & 1 \\ \end{array} } \right],{\mathbf{J}}_{{{\mathrm{q}},{\mathrm{Pl}},3}}^{1} = \left[ {\begin{array}{*{20}c} { - h \cdot {\mathrm{S}}q} & { - h \cdot {\mathrm{S}}q} \\ 0 & 0 \\ {h \cdot {\mathrm{C}}q - L} & {h \cdot {\mathrm{C}}q} \\ 0 & 0 \\ 1 & 1 \\ 0 & 0 \\ \end{array} } \right]. $$

For the right 3-RR Pl, the Jacobian matrices of its three chains can also be obtained in the same way.

### Kinematic modeling for the single limb (P(3-RR) mechanism)

In order to obtain the Jacobian matrices of each kinematic limb, it is necessary to analyze the kinematic transitions from point O_*i*_ to M_*i*_ through each limb, as shown in Fig. 3a. The kinematic homogenous matrix of limb 1 can be expressed as

21 $$ {\mathbf{T}}_{{{\mathrm{limb}}}}^{1} = {\mathbf{T}}_{{{\mathrm{base}}}}^{1} \cdot {\mathbf{T}}_{{{\mathrm{Ac}}}}^{1} \left( {\theta_{0}^{1} } \right) \cdot {\mathbf{T}}_{{{\mathrm{SP}}}}^{1} \cdot {\mathbf{T}}_{{{\mathrm{Vs}}}} \left( {\theta_{1}^{1} , \ldots ,\theta_{6}^{1} } \right) \cdot {\mathbf{T}}_{{{\mathrm{Pl}},1}}^{1} \cdot {\mathbf{T}}_{{{\mathrm{Vs}}}} \left( {\theta_{7}^{1} , \ldots ,\theta_{12}^{1} } \right) \cdot {\mathbf{T}}_{{{\mathrm{tool}}}}^{1} $$

where

$$ \begin{gathered}   {\mathbf{T}}_{{base}}^{1}  = \left[ {\begin{array}{*{20}c}    {{\mathbf{I}}_{3} } & {{\mathbf{P}}\left( {x, - L \cdot C_{q} } \right) + {\mathbf{P}}\left( {z, - L \cdot S_{q}  - l_{1}  - l_{2} } \right)}  \\    {\mathbf{0}} & 1  \\   \end{array} } \right],{\mathbf{T}}_{{Ac}}^{1} \left( {\theta _{{\mathbf{0}}}^{1} } \right) = \left[ {\begin{array}{*{20}c}    {{\mathbf{I}}_{3} } & {{\mathbf{P}}\left( {x,\theta _{{\mathbf{0}}}^{1} } \right)}  \\    {\mathbf{0}} & 1  \\   \end{array} } \right], \hfill \\   {\mathbf{T}}_{{SP}}^{1}  = \left[ {\begin{array}{*{20}c}    {{\mathbf{I}}_{3} } & {{\mathbf{P}}\left( {z,l_{1} } \right)}  \\    {\mathbf{0}} & 1  \\   \end{array} } \right],{\text{ }}{\mathbf{T}}_{{Vs}} \left( {\theta _{1}^{1} , \ldots ,\theta _{6}^{1} } \right) = \left[ {\begin{array}{*{20}c}    {{\mathbf{I}}_{3} } & {{\mathbf{P}}\left( {x,\theta _{1}^{1} } \right)}  \\    {\mathbf{0}} & 1  \\   \end{array} } \right] \cdot \left[ {\begin{array}{*{20}c}    {{\mathbf{I}}_{3} } & {{\mathbf{P}}\left( {y,\theta _{2}^{1} } \right)}  \\    {\mathbf{0}} & 1  \\   \end{array} } \right] \cdot \left[ {\begin{array}{*{20}c}    {{\mathbf{I}}_{3} } & {{\mathbf{P}}\left( {z,\theta _{3}^{1} } \right)}  \\    {\mathbf{0}} & 1  \\   \end{array} } \right] \hfill \\    \cdot \left[ {\begin{array}{*{20}c}    {{\mathbf{R}}\left( {x,\theta _{4}^{1} } \right)} & {\mathbf{0}}  \\    {\mathbf{0}} & 1  \\   \end{array} } \right] \cdot \left[ {\begin{array}{*{20}c}    {{\mathbf{R}}\left( {y,\theta _{5}^{1} } \right)} & {\mathbf{0}}  \\    {\mathbf{0}} & 1  \\   \end{array} } \right] \cdot \left[ {\begin{array}{*{20}c}    {{\mathbf{R}}\left( {z,\theta _{6}^{1} } \right)} & {\mathbf{0}}  \\    {\mathbf{0}} & 1  \\   \end{array} } \right],{\text{ }}{\mathbf{T}}_{{Pl,1}}^{1}  = \left[ {\begin{array}{*{20}c}    {{\mathbf{R}}\left( {y, - q} \right)} & {\mathbf{0}}  \\    {\mathbf{0}} & 1  \\   \end{array} } \right] \cdot \left[ {\begin{array}{*{20}c}    {{\mathbf{I}}_{3} } & {{\mathbf{P}}\left( {x,L} \right)}  \\    {\mathbf{0}} & 1  \\   \end{array} } \right], \hfill \\  \end{gathered}  $$

$$ \begin{gathered} {\mathbf{T}}_{{{\mathrm{Vs}}}} \left( {\theta_{7}^{1} ,...,\theta_{12}^{1} } \right) = \left[ {\begin{array}{*{20}c} {{\mathbf{I}}_{3} } & {{\mathbf{P}}\left( {x,\theta_{7}^{1} } \right)} \\ {\mathbf{0}} & 1 \\ \end{array} } \right] \cdot \left[ {\begin{array}{*{20}c} {{\mathbf{I}}_{3} } & {{\mathbf{P}}\left( {y,\theta_{8}^{1} } \right)} \\ {\mathbf{0}} & 1 \\ \end{array} } \right] \cdot \left[ {\begin{array}{*{20}c} {{\mathbf{I}}_{3} } & {{\mathbf{P}}\left( {z,\theta_{9}^{1} } \right)} \\ {\mathbf{0}} & 1 \\ \end{array} } \right] \cdot \left[ {\begin{array}{*{20}c} {{\mathbf{R}}\left( {x,\theta_{10}^{1} } \right)} & {\mathbf{0}} \\ {\mathbf{0}} & 1 \\ \end{array} } \right] \hfill \\ \cdot \left[ {\begin{array}{*{20}c} {{\mathbf{R}}\left( {y,\theta_{11}^{1} } \right)} & {\mathbf{0}} \\ {\mathbf{0}} & 1 \\ \end{array} } \right] \cdot \left[ {\begin{array}{*{20}c} {{\mathbf{R}}\left( {z,\theta_{12}^{1} } \right)} & {\mathbf{0}} \\ {\mathbf{0}} & 1 \\ \end{array} } \right], \, {\mathbf{T}}_{{{\mathrm{tool}}}}^{1} = \left[ {\begin{array}{*{20}c} {{\mathbf{R}}\left( {y,q} \right)} & {\mathbf{0}} \\ {\mathbf{0}} & 1 \\ \end{array} } \right] \cdot \left[ {\begin{array}{*{20}c} {{\mathbf{I}}_{3} } & {{\mathbf{P}}\left( {z,l_{2} } \right)} \\ {\mathbf{0}} & 1 \\ \end{array} } \right]. \hfill \\ \end{gathered} $$

The position and direction of the end point M_1_ can be extracted from the matrix ${\mathbf{T}}_{{{\mathrm{limb}}}}^{1}$ in a standard way, whereby the kinematic model can be rewritten in the form of a vector function as follows

22 $$ {\mathbf{t}}_{{{\mathrm{limb}}}}^{1} = {\mathbf{g}}\left( {{{\varvec{\uptheta}}}_{{{\mathrm{limb}}}}^{1} } \right) $$

where ${\mathbf{t}}_{{{\mathrm{limb}}}}^{1}$ is a vector that describes the position and direction of the end point M_1_, ${{\varvec{\uptheta}}}_{{{\mathrm{limb}}}}^{1}$ is a vector collecting all virtual spring coordinates ($\theta_{0}^{1} ,\theta_{1}^{1} ,...,\theta_{12}^{1}$).

According to the principle of virtual work, the virtual displacement $\delta {\mathbf{t}}_{{{\mathrm{limb}}}}^{1}$ can be computed from the linearized geometrical model:

23 $$ \delta {\mathbf{t}}_{{{\mathrm{limb}}}}^{1} = {\mathbf{J}}_{{{\uptheta },{\mathrm{limb}}}}^{1} \cdot \delta {{\varvec{\uptheta}}}_{{{\mathrm{limb}}}}^{1} $$

which includes the Jacobian matrix:

24 $$ {\mathbf{J}}_{{{\uptheta },{\mathrm{limb}}}}^{1} = \frac{\partial }{{\partial {{\varvec{\uptheta}}}_{{{\mathrm{limb}}}}^{1} }}{\mathbf{g}}\left( {{{\varvec{\uptheta}}}_{{{\mathrm{limb}}}}^{1} } \right) = \left[ {{\mathbf{J}}_{{{\uptheta },{\mathrm{limb}},0}}^{1} ,{\mathbf{J}}_{{{\uptheta },{\mathrm{limb}},1}}^{1} , \ldots ,{\mathbf{J}}_{{{\uptheta },{\mathrm{limb}},12}}^{1} } \right] $$

where

$$ \begin{gathered} \frac{\partial }{{\partial \theta_{n}^{1} }}{\mathbf{T}}_{{{\mathrm{limb}}}}^{1} = \left[ {\begin{array}{*{20}c} {{\mathbf{s}}\left( {{{\varvec{\upomega}}}_{{{\uptheta },{\mathrm{limb}},n}}^{1} } \right)} & {{\mathbf{p}}_{{{\uptheta },{\mathrm{limb}},n}}^{1} } \\ {\mathbf{0}} & 0 \\ \end{array} } \right], \, {\mathbf{J}}_{{{\uptheta },{\mathrm{limb}},n}}^{1} = \frac{\partial }{{\partial \theta_{n}^{1} }}{\mathbf{g}}\left( {{{\varvec{\uptheta}}}_{{{\mathrm{limb}}}}^{1} } \right) = \left[ {\begin{array}{*{20}c} {{\mathbf{p}}_{{{\uptheta },{\mathrm{limb}},n}}^{1} } \\ {{{\varvec{\upomega}}}_{{{\uptheta },{\mathrm{limb}},n}}^{1} } \\ \end{array} } \right], \hfill \\ {\mathbf{J}}_{{{\uptheta },{\mathrm{Ac}}}}^{1} = {\mathbf{J}}_{{{\uptheta },{\mathrm{limb}},0}}^{1} , \, {\mathbf{J}}_{{{\uptheta },{\mathrm{SP}}}}^{1} = \left[ {{\mathbf{J}}_{{{\uptheta },{\mathrm{limb}},1}}^{1} , \ldots ,{\mathbf{J}}_{{{\uptheta },{\mathrm{limb}},6}}^{1} } \right], \, {\mathbf{J}}_{{{\uptheta },{\mathrm{Pl}}}}^{1} = \left[ {{\mathbf{J}}_{{{\uptheta },{\mathrm{limb}},7}}^{1} , \ldots ,{\mathbf{J}}_{{{\uptheta },{\mathrm{limb}},12}}^{1} } \right], \hfill \\ \end{gathered} $$

${\mathbf{p}}_{{{\uptheta },{\mathrm{limb}},n}}^{1}$ denote the position vector associated with $\theta_{n}^{1}{^{\prime}}$, ${\mathbf{s}}\left( {{{\varvec{\upomega}}}_{{{\uptheta },{\mathrm{limb}},n}}^{1} } \right)$ is represents the skew-matrix of the orientation vector ${{\varvec{\upomega}}}_{{{\uptheta },{\mathrm{limb}},n}}^{1}$.

According to the above theory, the Jacobian matrices of limb 1 can be obtained by deriving Eq. (21) as follows:

25a $$ {\mathbf{J}}_{{{\uptheta },{\mathrm{Ac}}}}^{1} = \left[ {\begin{array}{*{20}c} 1 & 0 & 0 & 0 & 0 & 0 \\ \end{array} } \right]^{{\mathrm{T}}} , $$

25b $$ {\mathbf{J}}_{{{\uptheta },{\mathrm{SP}}}}^{1} = \left[ {\begin{array}{*{20}c} 1 & 0 & 0 & 0 & {L \cdot {\mathrm{S}}q + l_{2} } & 0 \\ 0 & 1 & 0 & { - L \cdot {\mathrm{S}}q - l_{2} } & 0 & {L \cdot {\mathrm{C}}q} \\ 0 & 0 & 1 & 0 & { - L \cdot {\mathrm{C}}q} & 0 \\ 0 & 0 & 0 & 1 & 0 & 0 \\ 0 & 0 & 0 & 0 & 1 & 0 \\ 0 & 0 & 0 & 0 & 0 & 1 \\ \end{array} } \right], $$

25c $$ {\mathbf{J}}_{{{\uptheta },{\mathrm{Pl}}}}^{1} = \left[ {\begin{array}{*{20}c} {{\mathrm{C}}q} & 0 & { - {\mathrm{S}}q} & 0 & {l_{2} } & 0 \\ 0 & 1 & 0 & { - l_{2} \cdot {\mathrm{C}}q} & 0 & {l_{2} \cdot {\mathrm{S}}q} \\ {{\mathrm{S}}q} & 0 & {{\mathrm{C}}q} & 0 & 0 & 0 \\ 0 & 0 & 0 & {{\mathrm{C}}q} & 0 & { - {\mathrm{S}}q} \\ 0 & 0 & 0 & 0 & 1 & 0 \\ 0 & 0 & 0 & {{\mathrm{S}}q} & 0 & {{\mathrm{C}}q} \\ \end{array} } \right]. $$

For limb 2, the Jacobian matrices of its three components can also be obtained in the same way.

### Kinematic modeling for the whole robot (P[2-P(3-RR)] mechanism)

The Biglide parallel robot is composed of a lead screw system (prismatic joint) capable of producing translation along the y_O_-axis in series with a 2-P(3-RR) parallel structure, where the 2-P(3-RR) parallel structure includes two limbs, a screw system capable of producing translation along the x_O_-axis, and a mobile platform, as shown in Fig. 2. In order to calculate the stiffness of the whole robot, the kinematic Jacobian matrices of the mobile platform and the prismatic joint need to be evaluated. These Jacobian matrices can be expressed as

26 $$ {\mathbf{J}}_{{\mathrm{v}}}^{i} = \left[ {\begin{array}{*{20}c} {{\mathbf{I}}_{3} } & {{\mathbf{s}}\left( {{\mathbf{r}}_{{\mathrm{v}}}^{i} } \right)} \\ {\mathbf{0}} & {{\mathbf{I}}_{3} } \\ \end{array} } \right], \, {\mathbf{r}}_{{\mathrm{v}}}^{1} = \left[ {\begin{array}{*{20}c} l \\ 0 \\ 0 \\ \end{array} } \right], \, {\mathbf{r}}_{{\mathrm{v}}}^{2} = \left[ {\begin{array}{*{20}c} { - l} \\ 0 \\ 0 \\ \end{array} } \right], \, {\mathbf{J}}_{{{\uptheta },{\mathrm{Ac}}}}^{0} = \left[ {\begin{array}{*{20}c} 0 & 1 & 0 & 0 & 0 & 0 \\ \end{array} } \right]^{{\mathrm{T}}} $$

where ${\mathbf{r}}_{{\mathrm{v}}}^{i}$ is the position vector from point M_*i*_ to point M expressed in the coordinate system {M}, ${\mathbf{s}}\left( {{\mathbf{r}}_{{\mathrm{v}}}^{i} } \right)$ represents the skew-symmetric matrix corresponding to the vector ${\mathbf{r}}_{{\mathrm{v}}}^{i}$^35,36^.

## Numerical analysis

**Figure 4:**
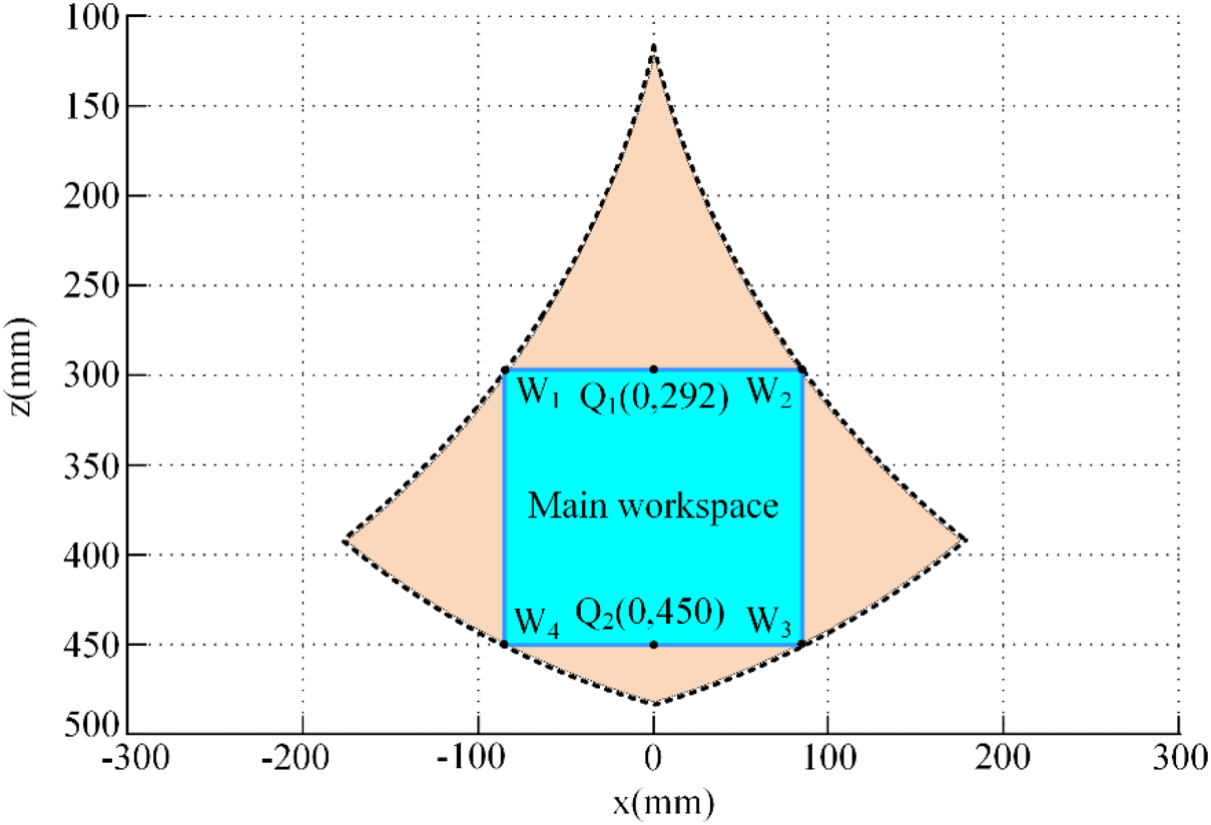
The reachable and regular workspace of the Biglide parallel robot.

In this section, the principal stiffness of the Biglide parallel robot considering the gravity of the mobile platform and the links is investigated numerically using the developed stiffness model. The dimensional and mass parameters of the robot are shown in Table 1. The position parameters of each limb are shown in Tables 2 and 3. The dimensional parameters of the link are shown in Table 4. The reachable workspace of Biglide parallel robot in the *x*–*z* plane is the yellow area in Fig. 4, and the blue area enclosed by W_1_-W_4_ is its main workspace, where Q_1_ and Q_2_ are the midpoints of W_1_W_2_ and W_3_W_4_, respectively.

**Table 1:** Parameters of the Biglide parallel mechanism.

*L* (mm)	*l* (mm)	*l*_1_ (mm)	*l*_2_ (mm)	*h* (mm)	*d* (mm)	*m*_link_ (kg)	*m*_MP_ (kg)	$K_{{{\uptheta },{\mathrm{Ac}}}}$ (N/m)
500	75	40	15	17.5	70	1.95	7.93	1.27·10^7^

**Table 2:** Location vector of the left limb (mm).

${\mathbf{r}}_{{{\mathrm{M}}1}}^{{\mathrm{M}}}$	${\mathbf{r}}_{{{\mathrm{B}}11}}^{{{\mathrm{M}}1}}$	${\mathbf{r}}_{{{\mathrm{B}}12}}^{{{\mathrm{M}}1}}$	${\mathbf{r}}_{{{\mathrm{B}}13}}^{{{\mathrm{M}}1}}$	${\mathbf{r}}_{{{\mathrm{G}}1j}}^{{{\mathrm{B}}1j}}$	${\mathbf{r}}_{{{\mathrm{A}}1j}}^{{{\mathrm{B}}1j}}$	${\mathbf{r}}_{{{\mathrm{O}}1}}^{{{\mathrm{M}}1}}$
$$\left[ {\begin{array}{*{20}c} { - l} \\ 0 \\ 0 \\ \end{array} } \right]$$	$$\left[ {\begin{array}{*{20}c} { - h} \\ 0 \\ { - l_{2} } \\ \end{array} } \right]$$	$$\left[ {\begin{array}{*{20}c} h \\ d \\ { - l_{2} } \\ \end{array} } \right]$$	$$\left[ {\begin{array}{*{20}c} h \\ { - d} \\ { - l_{2} } \\ \end{array} } \right]$$	$$\left[ {\begin{array}{*{20}c} { - {L \mathord{\left/ {\vphantom {L 2}} \right. \kern-0pt} 2}} \\ 0 \\ 0 \\ \end{array} } \right]$$	$$\left[ {\begin{array}{*{20}c} { - L} \\ 0 \\ 0 \\ \end{array} } \right]$$	$$\left[ {\begin{array}{*{20}c} { - L \cdot {\mathrm{C}}_{q} } \\ 0 \\ { - L \cdot {\mathrm{S}}_{q} - l_{1} - l_{2} } \\ \end{array} } \right]$$

**Table 3:** Location vector of the right limb (mm).

${\mathbf{r}}_{{{\mathrm{M}}2}}^{{\mathrm{M}}}$	${\mathbf{r}}_{{{\mathrm{B}}21}}^{{{\mathrm{M}}2}}$	${\mathbf{r}}_{{{\mathrm{B}}22}}^{{{\mathrm{B}}2}}$	${\mathbf{r}}_{{{\mathrm{B}}23}}^{{{\mathrm{B}}2}}$	${\mathbf{r}}_{{{\mathrm{G}}2j}}^{{{\mathrm{B}}2j}}$	${\mathbf{r}}_{{{\mathrm{A}}2j}}^{{{\mathrm{B}}2j}}$	${\mathbf{r}}_{{{\mathrm{A}}2}}^{{{\mathrm{B}}2}}$
$$\left[ {\begin{array}{*{20}c} l \\ 0 \\ 0 \\ \end{array} } \right]$$	$$\left[ {\begin{array}{*{20}c} h \\ 0 \\ { - l_{2} } \\ \end{array} } \right]$$	$$\left[ {\begin{array}{*{20}c} { - h} \\ d \\ { - l_{2} } \\ \end{array} } \right]$$	$$\left[ {\begin{array}{*{20}c} { - h} \\ { - d} \\ { - l_{2} } \\ \end{array} } \right]$$	$$\left[ {\begin{array}{*{20}c} { - {L \mathord{\left/ {\vphantom {L 2}} \right. \kern-0pt} 2}} \\ 0 \\ 0 \\ \end{array} } \right]$$	$$\left[ {\begin{array}{*{20}c} { - L} \\ 0 \\ 0 \\ \end{array} } \right]$$	$$\left[ {\begin{array}{*{20}c} {L \cdot {\mathrm{C}}_{q} } \\ 0 \\ { - L \cdot {\mathrm{S}}_{q} - l_{1} - l_{2} } \\ \end{array} } \right]$$

**Table 4:** Parameters of the cylindrical link.

*L* (mm)	*r* (mm)	*E* (GPa)	*G* (GPa)
500	12.5	211	81

### Compliance matrices

The 3-RR Pl contains three identical cylindrical links, as shown in Fig. 3. In order to calculate the Cartesian stiffness matrix of the 3-RR Pl, it is necessary to obtain the compliance matrix of each link by means of the cantilever beam principle^16,19^. Its compliance matrix ${\mathbf{K}}_{{{\uptheta },{\mathrm{Pl}},j}}^{i \, - 1}$ can be expressed as

28 $$ {\mathbf{K}}_{{{\uptheta },{\mathrm{Pl}},j}}^{i \, - 1} = \left[ {\begin{array}{*{20}c} {\tfrac{L}{EA}} & 0 & 0 & 0 & 0 & 0 \\ 0 & {\tfrac{{L^{3} }}{{3EI_{z} }}} & 0 & 0 & 0 & {\tfrac{{L^{2} }}{{2EI_{z} }}} \\ 0 & 0 & {\tfrac{{L^{3} }}{{3EI_{y} }}} & 0 & { - \tfrac{{L^{2} }}{{2EI_{y} }}} & 0 \\ 0 & 0 & 0 & {\tfrac{L}{{GI_{p} }}} & 0 & 0 \\ 0 & 0 & { - \tfrac{{L^{2} }}{{2EI_{y} }}} & 0 & {\tfrac{L}{{EI_{y} }}} & 0 \\ 0 & {\tfrac{{L^{2} }}{{2EI_{z} }}} & 0 & 0 & 0 & {\tfrac{L}{{EI_{z} }}} \\ \end{array} } \right] $$

where *I*_*y*_, *I*_*z*_ and *I*_*p*_ are the quadratic and polar moments of inertia of the cross-section, and *E* and *G* are the Young’s and shear modules, respectively.

Moreover, the slidable platform stiffness can be identified on the basis of CAD models and FEA methods^10,25,37^. Its compliance matrix ${\mathbf{K}}_{{{\uptheta },{\mathrm{SP}}}}^{i \, - 1}$ can be expressed as (unit: N, m, rad)

29 $$ {\mathbf{K}}_{{{\uptheta },{\mathrm{SP}}}}^{i \, - 1} = \left[ {\begin{array}{*{20}c} {0.063} & 0 & 0 & 0 & 0 & 0 \\ 0 & {0.018} & 0 & 0 & 0 & {0.313} \\ 0 & 0 & {4.548} & 0 & { - 80.255} & 0 \\ 0 & 0 & 0 & {19.140} & 0 & 0 \\ 0 & 0 & { - 80.255} & 0 & {1888.421} & 0 \\ 0 & {0.313} & 0 & 0 & 0 & {7.376} \\ \end{array} } \right] \times 10^{ - 9} . $$

Finally, the Cartesian stiffness matrix ${\mathbf{K}}_{{\mathrm{C}}}^{{\mathrm{O}}}$ of the whole robot in the coordinate system {O} can be obtained by a stiffness matrix transformation^9,33^. The Cartesian stiffness matrices ${\mathbf{K}}_{{{\mathrm{C}},{\mathrm{P1}}}}^{{\mathrm{O}}}$ and ${\mathbf{K}}_{{{\mathrm{C}},{\mathrm{P2}}}}^{{\mathrm{O}}}$ at the points P1 and P2 can respectively be expressed as (unit: N, m, rad)

30a $$ {\mathbf{K}}_{{{\mathrm{C}},{\mathrm{P1}}}}^{{\mathrm{O}}} = \left[ {\begin{array}{*{20}c} {21.862} & 0 & 0 & 0 & { - 1.981} & 0 \\ 0 & {1.969} & 0 & {0.275} & 0 & { - 0.004} \\ 0 & 0 & {4015.669} & 0 & {7.810} & 0 \\ 0 & {0.275} & 0 & {1.500} & 0 & {0.001} \\ { - 1.981} & 0 & {7.810} & 0 & {17.337} & 0 \\ 0 & { - 0.004} & 0 & {0.001} & 0 & {3.079} \\ \end{array} } \right] \times 10^{6} , $$

30b $$ {\mathbf{K}}_{{{\mathrm{C}},{\mathrm{P2}}}}^{{\mathrm{O}}} = \left[ {\begin{array}{*{20}c} {11.788} & 0 & 0 & 0 & { - 0.370} & 0 \\ 0 & {1.969} & 0 & {0.427} & 0 & { - 0.009} \\ 0 & 0 & {1659.962} & 0 & {7.810} & 0 \\ 0 & {0.427} & 0 & {3.402} & 0 & {0.001} \\ { - 0.370} & 0 & {7.810} & 0 & {7.406} & 0 \\ 0 & { - 0.009} & 0 & {0.001} & 0 & {0.936} \\ \end{array} } \right] \times 10^{6} . $$

### Distribution of the principal stiffness

**Figure 5:**
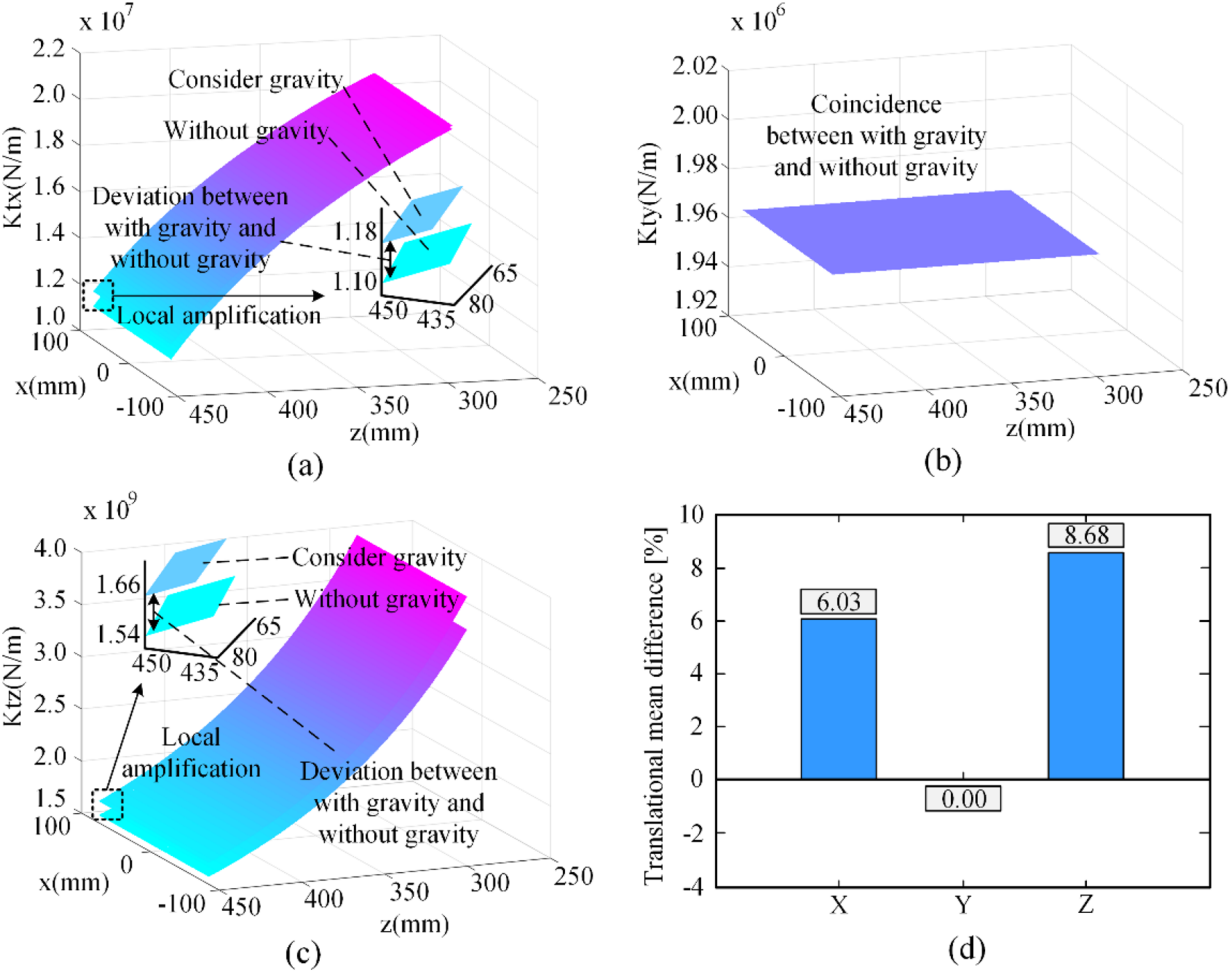
Principal translational stiffness distributions.

**Figure 6:**
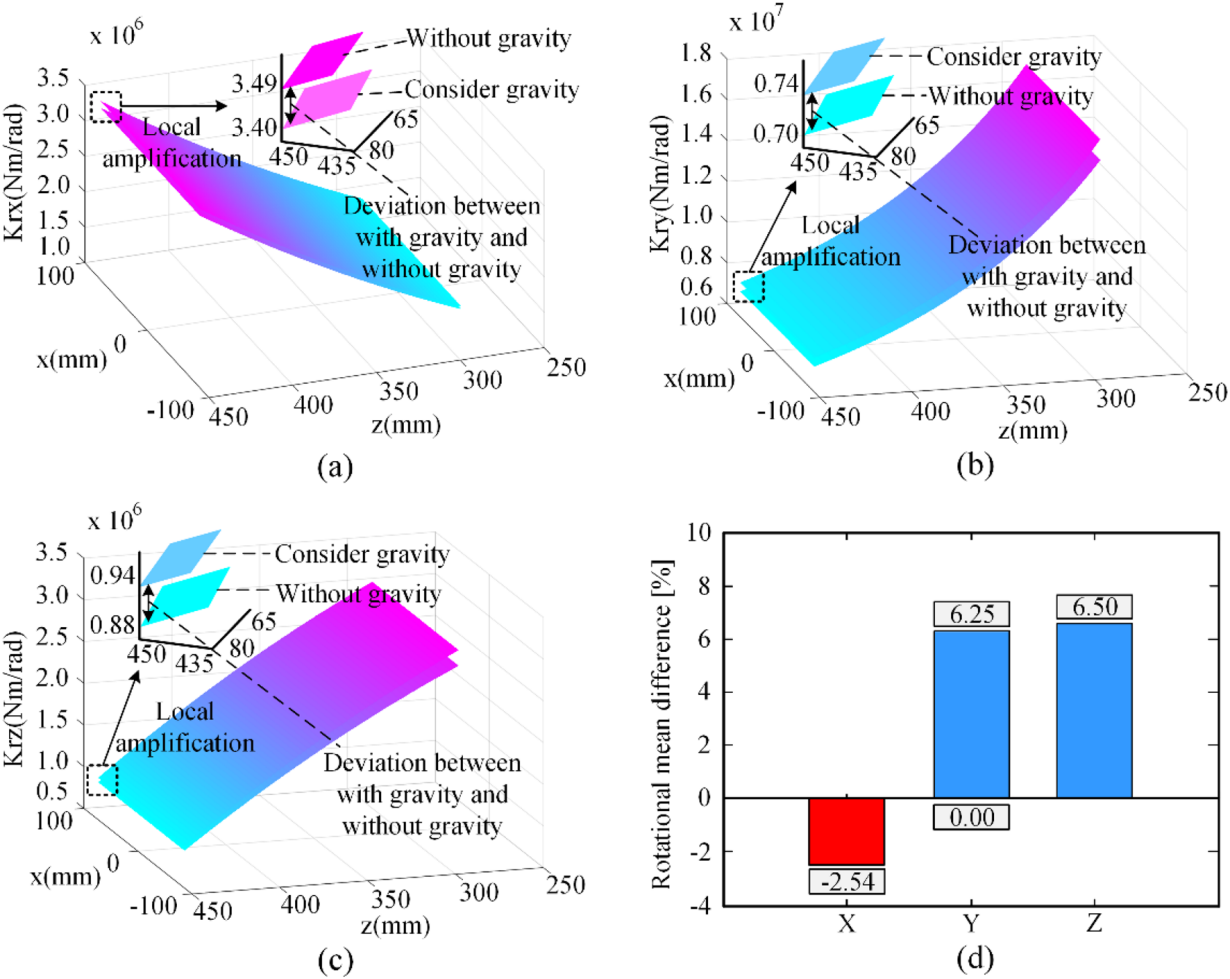
Principal rotational stiffness distributions.

In order to better evaluate the Cartesian stiffness of the Biglide parallel robot, we visualize the distribution of its principal stiffness in six directions in its workspace, as shown in Figs. 5 and 6. K_tx_, K_ty_, K_tz_ and K_rx_, K_ry_, K_rz_ are the diagonal elements of the Cartesian stiffness matrix ${\mathbf{K}}_{{\mathrm{C}}}^{{\mathrm{O}}}$, which represent the principal translational stiffness along the x, y, z axes and the principal rotational stiffness around the x, y, z axes, respectively.

Figure 5a shows the principal translational stiffness K_tx_ along the x-axis with and without gravity, it can be seen that the stiffness with gravity improves compared to the stiffness without gravity, and they both decrease as the z-axis value increases; Fig. 5b shows the principal translational stiffness K_ty_ along the y-axis with and without gravity, it is obvious that the stiffness with gravity is the same as the stiffness without gravity, and this stiffness is a constant; Fig. 5c shows the principal translational stiffness K_tz_ along the z-axis with and without gravity, it has similar stiffness characteristics to K_tx_; and Fig. 5d shows the mean difference between the principal translational stiffness with and without gravity. Moreover, among the principal translational stiffness K_tz_ is the highest order of magnitude translational stiffness, K_tx_ is the second and K_ty_ is the smallest.

Figure 6 is similar to Fig. 5, but the difference is that Fig. 6 shows the principal rotational stiffness with and without gravity and their mean difference. It is worth noting that the stiffness K_rx_ in Fig. 6a becomes smaller due to the gravitational influence and it is the only principal stiffness where gravity has a negative effect. K_rx_ increases with increasing z-axis values, while K_ry_ and K_rz_ decrease with increasing z-axis values. Furthermore, K_ry_ is the highest order of magnitude rotational stiffness in the principal rotational stiffness, and K_rx_ and K_rz_ have the same order of magnitude.

## Experimental analysis

**Figure 7:**
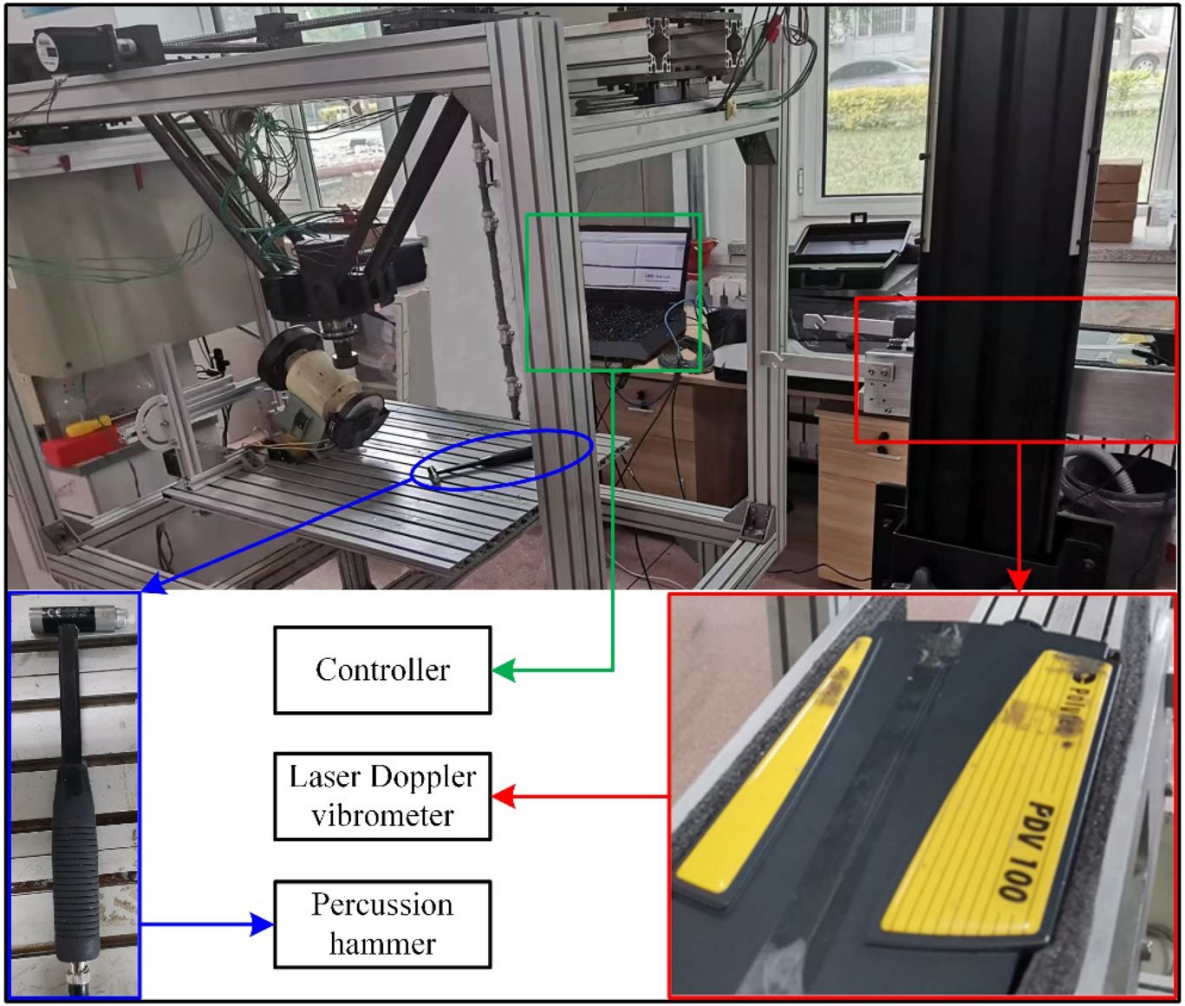
The knocking experimental device.

To validate the correctness of the stiffness model with gravity, the knocking experiments were employed on the Biglide industrial parallel robot. Using a laser Doppler vibrometer as the main experimental equipment, the principal stiffness of the mobile platform center of the Biglide parallel robot was measured in all directions, as shown in Fig. 7.

**Figure 8:**
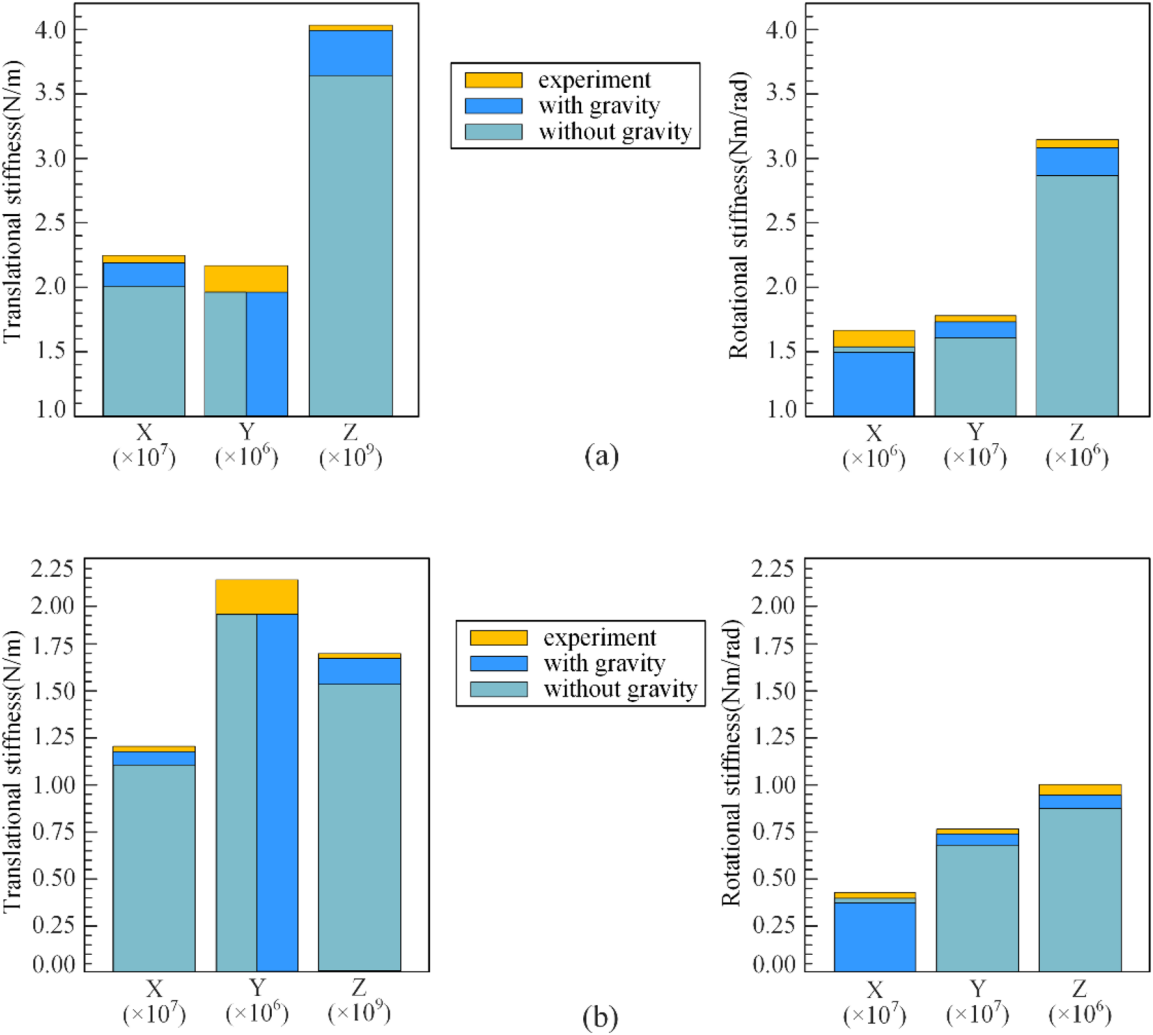
Principal stiffness: (**a**) at P1 point; (**b**) at P2 point.

Table 5 lists the principal stiffness in each direction at point Q_1_. The values at point Q_2_ are listed in Table 6. These values are presented in a more intuitive form in Fig. 8. It can be found that the principal stiffness of the stiffness model with gravity is very close to the experimental results. The root mean square (RMS) values between the developed model and the experimental results at points Q_1_ and Q_2_ are 2.67 and 2.77%, respectively^38,39^. And the RMS values between the stiffness model without gravity and the experimental results at Q_1_ and Q_2_ are 9.043 and 9.353%, respectively. The experimental results show a good agreement with the theoretical values of the ‘with gravity’ model. Therefore, it can be concluded that the stiffness model with gravity is effective, which lays a solid foundation for the real applications.

**Table 5:** Principal stiffness at Q_1_.

Model	K_tx_ (× 10^6^) (N/m)	K_ty_ (× 10^6^) (N/m)	K_tz_ (× 10^9^) (N/m)	K_rx_ (× 10^6^) (Nm/rad)	K_ry_ (× 10^6^) (Nm/rad)	K_rz_ (× 10^6^) (Nm/rad)
Without gravity	20.664	1.969	3.684	1.538	16.279	2.883
With gravity	21.862	1.969	4.016	1.500	17.337	3.079
Experiment	22.568	2.198	4.039	1.687	17.888	3.155

**Table 6:** Principal stiffness at Q_2_.

Model	K_tx_ (× 10^6^) (N/m)	K_ty_ (× 10^6^) (N/m)	K_tz_ (× 10^9^) (N/m)	K_rx_ (× 10^6^) (Nm/rad)	K_ry_ (× 10^6^) (Nm/rad)	K_rz_ (× 10^6^) (Nm/rad)
Without gravity	11.096	1.969	1.537	3.493	6.987	0.881
With gravity	11.788	1.969	1.660	3.402	7.406	0.936
Experiment	12.018	2.194	1.682	3.807	7.640	1.001

## Conclusions

This paper proposes a novel stiffness modeling method for an industrial parallel robot, which is important for the machining of parallel robots. The main research results are summarized as follows:

1. The influences of the gravity of the movable links, the gravity of the mobile platform and their corresponding mass centers are considered simultaneously. In order to obtain the values of gravity, the mathematical equations of the static model were established for each joint.
2. The stiffness model of each component is established by considering its compliance matrix, the compliance of the corresponding joint, its Jacobian matrix from the corresponding mathematical equations of the kinematic model, and the corresponding stiffness matrix due to external gravity. For the accuracy of the calculation, the compliance matrix of the irregular component is identified by using the FEA-based virtual experiment.
3. The validity of the stiffness model with gravity was verified by comparing the calculated stiffness of the Biglide industrial parallel robot with the knockout experiment. The results show that the method is able to obtain sufficient computational accuracy to predict the stiffness distribution in the task workspace. This enables the robot to obtain more accurate tool head trajectory before machining, thus improving its machining accuracy.
4. The stiffness modeling method is also applicable to the stiffness estimation of the robot structure design stage, which provides effective help for the improvement and verification of the structure.

## Data Availability

The datasets generated and/or analysed during the current study are not publicly available due the data also forms part of an ongoing study but are available from the corresponding author on reasonable request.
